# Photo-induced high-temperature ferromagnetism in YTiO_3_

**DOI:** 10.1038/s41586-023-05853-8

**Published:** 2023-05-03

**Authors:** A. S. Disa, J. Curtis, M. Fechner, A. Liu, A. von Hoegen, M. Först, T. F. Nova, P. Narang, A. Maljuk, A. V. Boris, B. Keimer, A. Cavalleri

**Affiliations:** 1 Max Planck Institute for the Structure and Dynamics of Matter, Hamburg, Germany; 2 School of Applied and Engineering Physics, Cornell University, Ithaca, NY USA; 3 John A. Paulson School of Engineering and Applied Sciences, Harvard University, Cambridge, MA USA; 4 College of Letters and Science, University of California, Los Angeles, CA USA; 5 Leibniz Institute for Solid State and Materials Research Dresden, Dresden, Germany; 6 Max Planck Institute for Solid State Research, Stuttgart, Germany; 7 Clarendon Laboratory, Department of Physics, Oxford University, Oxford, UK

**Keywords:** Ferromagnetism, Phase transitions and critical phenomena, Terahertz optics, Ultrafast photonics, Magnetic properties and materials

## Abstract

In quantum materials, degeneracies and frustrated interactions can have a profound impact on the emergence of long-range order, often driving strong fluctuations that suppress functionally relevant electronic or magnetic phases^1–7^. Engineering the atomic structure in the bulk or at heterointerfaces has been an important research strategy to lift these degeneracies, but these equilibrium methods are limited by thermodynamic, elastic and chemical constraints^8^. Here we show that all-optical, mode-selective manipulation of the crystal lattice can be used to enhance and stabilize high-temperature ferromagnetism in YTiO_3_, a material that shows only partial orbital polarization, an unsaturated low-temperature magnetic moment and a suppressed Curie temperature, *T*_c_ = 27 K (refs. ^9–13^). The enhancement is largest when exciting a 9 THz oxygen rotation mode, for which complete magnetic saturation is achieved at low temperatures and transient ferromagnetism is realized up to *T*_neq_ > 80 K, nearly three times the thermodynamic transition temperature. We interpret these effects as a consequence of the light-induced dynamical changes to the quasi-degenerate Ti *t*_2g_ orbitals, which affect the magnetic phase competition and fluctuations found in the equilibrium state^14–20^. Notably, the light-induced high-temperature ferromagnetism discovered in our work is metastable over many nanoseconds, underscoring the ability to dynamically engineer practically useful non-equilibrium functionalities.

## Abstract (web summary)

All-optical, mode-selective manipulation of the crystal lattice can be used to enhance and stabilize ferromagnetism in YTiO_3_ well above its equilibrium ordering temperature and for many nanoseconds, enabling dynamic engineering of practically useful non-equilibrium functionalities in fluctuating electronic systems.

## Main

The macroscopic properties of quantum materials are determined by a delicate tension between microscopic elements, the most relevant being the crystal structure, the magnetic state of the constituent electrons and the orbitals that they occupy. Degeneracies and their lifting play a fundamental role. For instance, Jahn–Teller distortions lift orbital degeneracies in certain correlated insulators and lead to the stabilization of long-ranged spin and orbital order^3,4^. In cases where degeneracies are not effectively lifted, long-range electronic order is often depressed and precursor fluctuations are observed far above the thermodynamic transition temperature^1,2^. In particular, recent work has highlighted the key role of the orbital configuration in determining the stability of superconducting, magnetic and other electronically ordered phases of correlated materials^5–8^.

**Fig. 1:**
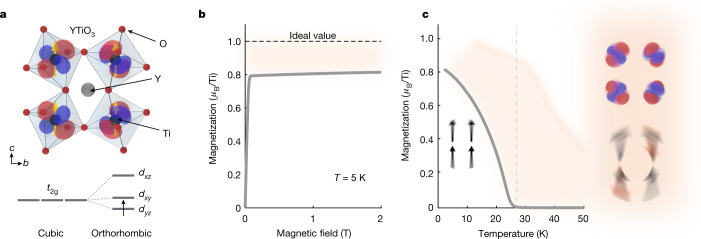
Fluctuating spin–orbital order in YTiO_3_. **a**, Crystal structure along with the associated low-temperature ferromagnetic and orbital ordering pattern. The orthorhombic structure determines the crystal field splitting and orbital mixing of the Ti *t*_2g_ levels on each Ti site. **b**, Magnetization as a function of magnetic field measured at *T* << *T*_c_, which saturates at high fields to roughly 0.8 *μ*_B_ per Ti, well below the theoretical limit. Fluctuations of the lattice and orbitals weaken ferromagnetic order through competing antiferromagnetic interactions, manifesting as a diminished magnetic moment and reduced critical temperature. **c**, Magnetization as a function of temperature. Spin correlations extend well above *T*_c_ = 27 K. The inset schematically shows the fluctuating orbital and spin configurations within the shaded region above *T*_c_. Source Data

One of the clearest examples of this behaviour is found in the family of Mott insulating rare-earth titanates (*R*TiO_3_)^9^. The low-energy physics of this system is dictated by a single Ti electron occupying a manifold of *d* orbitals with *t*_2g_ symmetry, namely the *d*_*xz*_, *d*_*yz*_ and *d*_*xy*_ orbitals (Fig. 1a). In the Goodenough–Kanamori–Anderson picture, the superexchange process is expected to favour antiferromagnetic or ferromagnetic spin interactions for electrons hopping between the same or orthogonal orbitals, respectively, leading to a strong dependence on the details of the bonding and crystal field environment at each Ti site. The ideal cubic perovskite structure with degenerate *t*_2g_ levels would create a highly frustrated ground state with large composite spin–orbital fluctuations^10,11^. In reality, Jahn–Teller and GdFeO_3_-type orthorhombic structural distortions lift the *t*_2g_ degeneracy in the titanates, pushing the system towards a static orbital ordering pattern and tipping the balance in favour of a particular magnetic state^21–25^.

Owing to this intricate balance of interactions, magnetism in the titanates is highly susceptible to small changes in crystal structure. For example, on decreasing the rare-earth ion size from *R* = La to *R* = Y (which increases the magnitude of structural distortions), one observes a crossover from an antiferromagnetic to a ferromagnetic ordered phase at low temperatures^14^. Moreover, numerous experimental studies show evidence for magnetic instabilities over a wide temperature range, which may be tied to fluctuations of the lattice and/or orbitals^15–19^.

In ferromagnetic YTiO_3_ (*T*_c_ ≅ 27 K), such fluctuations manifest in several ways. First, the critical temperature is considerably suppressed with respect to the mean field value (${T}_{{\rm{c}}}^{{\rm{mf}}}$ of roughly 50 K)^11,12^, and the magnetic moment at low temperatures is found to saturate well below the ideal spin-half limit, even for *T* << *T*_c_ (Fig. 1b)^13^. Second, magnetic contributions to the specific heat and thermal expansion are observed up to more than 3 × *T*_c_ (ref. ^20^). Third, anomalous phonon-frequency shifts attributable to spin correlations have been detected at similarly high temperatures (Extended Data Fig. 10).

The presence of these experimental signatures suggests that under equilibrium conditions, long-range ferromagnetic ordering in YTiO_3_ may be stifled by competing interactions or dynamical fluctuations. Such effects effectively limit the use of this and related correlated quantum materials for building new technologies. Thus, a question of key importance is whether, and by what means, it is possible to harness their harmful fluctuations to attain enhanced functional properties.

In this paper, we take advantage of the strong spin-lattice coupling of YTiO_3_ to address this problem, demonstrating the ability to enhance ferromagnetism with light. In particular, we resonantly excite vibrational modes of the lattice using intense terahertz frequency optical pulses. Deformations of the crystal structure not found in equilibrium become possible through the light–matter interaction, which can be engineered by selectively exciting specific phonons^26–28^. Previously, this technique has proved to be an effective tool to alter both local electronic states and their interactions in correlated materials as a means to modify their phases^29–34^, and we exploit it here to control orbital/magnetic order in YTiO_3_ through the lattice^35,36^.

## Experimental design

**Fig. 2:**
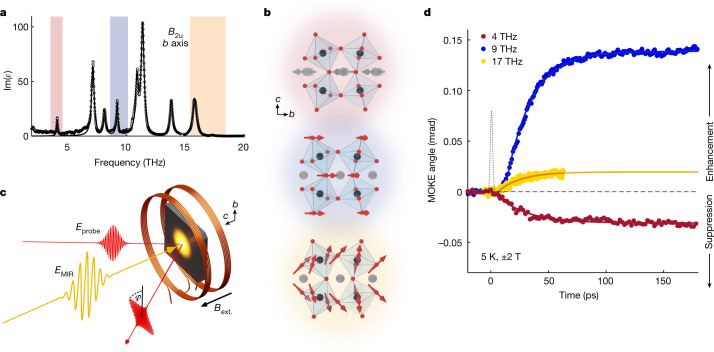
Phonon-selective control of ferromagnetism in YTiO_3_. **a**, Infrared optical spectrum of *b-*axis vibrational modes (black). The three phonons pumped in this experiment are shaded in red (4 THz), blue (9 THz) and yellow (17 THz). **b**, The eigen-displacements corresponding to the pumped vibrational modes in **a**: the low-frequency mode primarily involves antipolar motions of the Y ions, whereas the two higher-frequency modes mainly consist of displacements of the apical and equatorial oxygens, respectively, within the TiO_6_ octahedral cage. **c**, Depiction of the experimental set-up for the time-resolved MOKE measurements. *E*_MIR_, terahertz/mid-infrared pump; *E*_probe_, 800 nm probe; *B*_ext._, external magnetic field. **d**, Pump-induced changes of the MOKE angle (${\phi }_{{\rm{M}}}=\,\frac{\phi \left(+H\right)-\phi (-H)}{2}$) for the three different phonon excitations. The black dotted line shows the pump pulse envelope. Source Data

We restrict ourselves to *b-*axis modes with *B*_2u_ symmetry (Fig. 2a), which were estimated to have the strongest spin coupling from linear infrared spectroscopy. The coupling strength varies both in sign and magnitude across the nine *B*_2u_ modes (Extended Data Fig. 10 and Supplementary Fig. 1). For our experimental study, we focused on the modes with centre frequencies near 4, 9 and 17 THz, which are relatively well separated from other modes, have comparable oscillator strengths, and are predicted to show substantially different coupling to the spin sector (Methods). The atomic motions associated with these modes are shown in Fig. 2b.

The excitation pulses were generated using a recently developed terahertz source based on chirped pulse difference-frequency generation in an organic crystal, producing narrow bandwidth and high intensity pulses over the entire frequency range where phonon resonances are found, spanning far to mid-infrared wavelengths (Methods). The pulse durations range from roughly 150 fs for the highest frequency excitation to roughly 350 fs for the lowest, and the pump fluence was kept at 5 mJ cm^−^^2^ (corresponding to peak electric fields of roughly 2–4 MV cm^−1^) for all measurements, unless otherwise noted. (Note that the bandwidth of 17 THz excitation spans both of the highest modes in YTiO_3_. They are found to have similar effects on the magnetic properties, and our theoretical analysis takes into account the contributions of both modes.) For these field strengths, we estimate the oscillatory displacements for these modes to range between 5 and 10 pm, far above the rms thermal fluctuations of the equilibrium state (Supplementary Information).

To determine the changes to the magnetic state of YTiO_3_ induced by phonon excitation, we carried out time-resolved magneto-optic Kerr effect (MOKE) measurements. A schematic of the experimental pump-probe set-up is shown in Fig. 2c (more experimental details can be found in the Methods). The terahertz excitation pulses were focused at normal incidence to the (001) surface of a YTiO_3_ single crystal, propagating parallel to the ferromagnetic *c* axis, and linearly polarized along the *b* axis to excite the relevant *B*_2u_ modes. The MOKE signal was determined from the polarization rotation of a time-delayed probe pulse reflected from the sample as a function of external magnetic field (*H* ‖ *c*). In this geometry, the MOKE angle, *φ*_M_ (the component of the polarization rotation antisymmetric with respect *H*), is proportional to the *c-*axis magnetization (Extended Data Fig. 4).

## Phonon-selective control of magnetism

Figure 2d shows the pump-induced change in the MOKE angle, Δ*φ*_M_, as a function of time for each of the three excitation frequencies, taken at *T* = 5 K with *H* = ±2 T. The signal was calibrated such that positive Δ*φ*_M_ signifies an increase in the already existing ferromagnetic magnetization in equilibrium, whereas negative Δ*φ*_M_ signifies a reduction. For all cases, we observed that the pump initiated a gradual change in the magnetization that plateaued at a maximum value on a time scale of roughly 50 ps, after which it remained stable with no measurable decay through the duration of our measurement window (200 ps). We estimate from fitting data taken with longer time windows that the lifetime of this pump-induced state is at least several nanoseconds (Methods and Extended Data Fig. 9).

Notably, the sign and strength of the effect was found to differ significantly depending on the phonon being pumped. For the 4 THz pump, Δ*φ*_M_ was negative and relatively small (plateauing at −0.04 mrad), indicating that ferromagnetic order was weakened by the phonon excitation. On the other hand, the positive signal for the 9 and 17 THz modes indicates that driving these phonons enhanced the ferromagnetism of YTiO_3_. In addition, the 9 THz phonon was three times as effective in producing a change in magnetization as the other phonons. These results point to the existence of a long-lasting non-equilibrium state whose magnetic properties are highly sensitive to the structural distortions induced by the resonant THz drive.

**Fig. 3:**
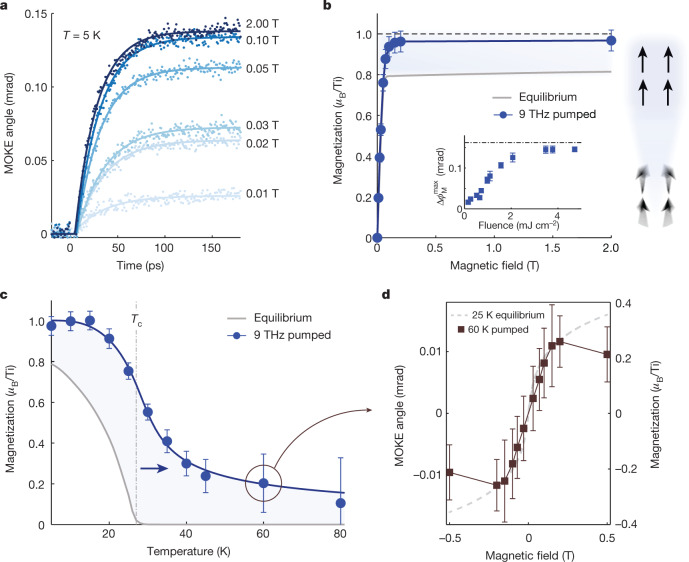
Characterization of the 9 THz pumped non-equilibrium state. **a**, Time-resolved MOKE signal for different magnetic fields at *T* = 5 K. **b**, Extracted maximum non-equilibrium magnetization (at *t* > 100 ps) (blue circles), compared to the equilibrium magnetization (grey solid line). The light-enhanced magnetic state nearly reaches the ideal spin-half limit (dotted line) suggesting a suppression of spin fluctuations. The inset shows the fluence dependence of the maximum pump-induced Δ*φ*_M_, which saturates at high fluence just below the limit. **c**, Temperature dependence of the non-equilibrium magnetization (blue circles). The light-induced effect extends up to at least 80 K, well above the equilibrium case (grey solid line). **d**, Maximum pump-induced MOKE signal as a function of magnetic field at *T* = 60 K. The field dependence follows that of equilibrium YTiO_3_ below *T*_c_, indicating the stabilization of a high-temperature ferromagnetic state. Error bars in **b**–**d** represent propagated uncertainty in *M*, as detailed in the Methods. Source Data

The magnetic field dependence of the time-resolved MOKE signal provides further insight into this phenomenon, as illustrated in a series of measurements examining the case of 9 THz phonon excitation. The time evolution of Δ*φ*_M_ is similar for all magnetic field strengths, but the maximum value attained at long time delays varies strongly (Fig. 3a). We can extract the *M*–*H* behaviour in the plateau region (*t* > 100 ps) by converting Δ*φ*_M_ into a magnetization using the calibration procedure outlined in the Methods. Figure 3b shows that, compared to equilibrium YTiO_3_, the saturation magnetization at high magnetic fields (*H* > 0.2 T) is roughly 20% greater in the non-equilibrium state, approaching 1 *μ*_B_ per Ti. Overall, the light-driven *M*–*H* dependence resembles that of ideal ferromagnetic YTiO_3_ absent competing interactions, hinting at the possibility that the phonon-mediated enhancement stems from a reduction in the fluctuations thought to suppress the ferromagnetic order in equilibrium. Validating this notion is the fluence dependence of the pump-induced MOKE signal, which saturates just below the 1 *μ*_B_ per Ti value for sufficiently large fluences (Fig. 3b, inset).

## Light-induced magnetism above *T*_c_

Having observed the enhancement in ferromagnetism well below the equilibrium Curie temperature, we investigated how this dynamical effect evolves as a function of temperature (Fig. 3c). Unlike for the unperturbed system, in which the magnetization drops to zero at *T*_c_, we found that pumping YTiO_3_ at 9 THz induced a magnetization up to temperatures in excess of 80 K—nearly three times *T*_c_—matching the temperature scale associated with anomalous magnetic correlations found in equilibrium YTiO_3_ (illustrated as orange region in Fig. 1c)^20^. The magnetic field dependence of the pump-induced signal above *T*_c_ shows a nonlinear *M–H* characteristic reminiscent to that at low temperatures but with a weaker spin stiffness (Fig. 3d). Hence, the phonon excitation creates a transient, non-equilibrium ferromagnetic state above *T*_c_ that persists to an effective onset temperature much higher than in equilibrium.

**Fig. 4:**
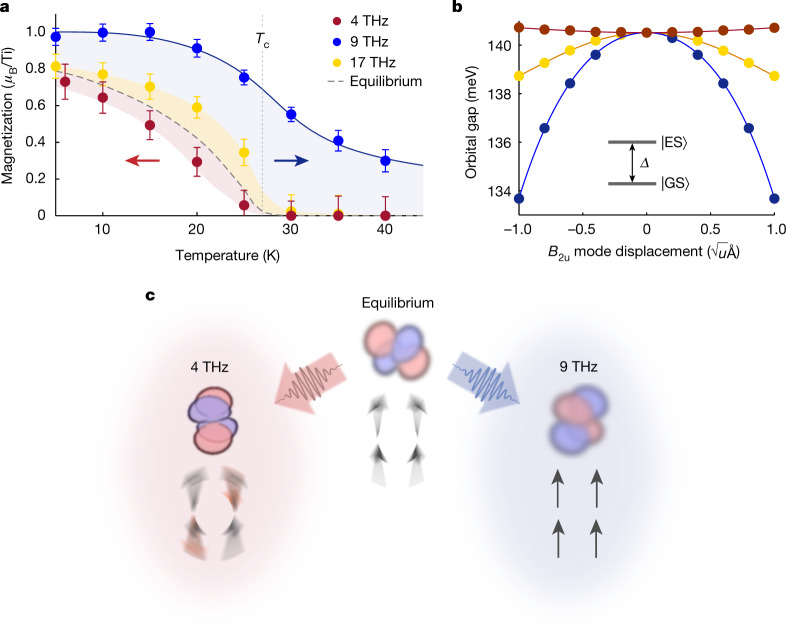
Possible origin of phonon-driven enhancement of ferromagnetism. **a**, Temperature dependence of non-equilibrium magnetization for each pump excitation frequency (coloured circles). Error bars represent propagated uncertainty in *M*, as detailed in the Methods. **b**, The energy gap *Δ* between the orbital ground state $| {\rm{GS}}\rangle$ and first excited orbital state $| {\rm{ES}}\rangle$ for each of the three excited phonons. The experimental mode displacements are on the order of $1\sqrt{u}{\rm{\mathring{\rm A} }}$ in magnitude (*u*, atomic mass units). **c**, Illustration of the equilibrium orbital ground state and the changes $({| {\rm{GS}}\rangle }_{{\rm{pumped}}}-{| {\rm{GS}}\rangle }_{{\rm{equil.}}})$ induced by driving the 4 THz and 9 THz phonons. We propose that the structural and orbital changes push the system farther or closer to the phase boundary, respectively, thereby suppressing or enhancing detrimental spin fluctuations. Source Data

As might be expected from the low-temperature behaviour, the non-equilibrium onset temperature *T*_neq_ depends on the phonon being pumped (Fig. 4a). The 17 THz phonon, which also showed an enhancement at low temperatures, produced a *T*_neq_ larger than *T*_c_ (but smaller than for 9 THz), whereas the 4 THz phonon, for which the low-temperature magnetic state was diminished, led to a state with *T*_neq_ slightly less than *T*_c_. The shift in the magnetic onset temperature relative to *T*_c_ roughly scales with the change in magnetization found at *T* << *T*_c_ (Fig. 2d).

## Discussion

On the basis of the stark differences in the pump-induced response when driving different vibrational modes, the origin of the observed magnetic behaviour must be linked to the associated coherent structural distortions. To investigate these effects, we start from frozen-phonon density functional theory (DFT) calculations, which are used to compute the spin–phonon coupling constants for each mode, ${\lambda }_{{\rm{spin}}-{\rm{ph}}}=\frac{1}{2\omega }\frac{{\partial }^{2}J}{{\partial }^{2}Q}$, where *Q* is the phonon amplitude, *ω* is the phonon frequency and *J* is the exchange interaction (Supplementary Information). The calculated *λ*_spin-ph_ values agree well with those determined experimentally from equilibrium infrared spectroscopy (Extended Data Fig. 10). When considering large phonon amplitudes, one expects an average change of the exchange interaction $\Delta J=\omega {\lambda }_{{\rm{s}}{\rm{p}}{\rm{i}}{\rm{n}}-{\rm{p}}{\rm{h}}}{Q}^{2}$, potentially explaining the observed pump-induced, non-equilibrium response; however, evaluating Δ*J* under the phonon drive yields predictions with the opposite sign compared to the experiment (discussed in the Supplementary Information). This conclusion does not depend on the value of the Hubbard *U* parameter used in the DFT calculations (Supplementary Information). This discrepancy suggests that physics beyond a naïve, adiabatic spin–phonon coupling are needed to explain the experimental results.

As noted in the introduction, the rare-earth titanates, and especially YTiO_3_, show strong fluctuations derived from competing spin–orbital–lattice interactions^9,11–13,16–20^. The relevant physics can be theoretically treated using a Kugel–Khomskii type Hamiltonian^11,21,37^. The ground state wavefunction in this model, which depends critically on displacements of the Y and O ions, dictates the allowed superexchange pathways and the relative energies of the various types of magnetic order. For equilibrium YTiO_3_, it has been found that the orbital ground state (that is, the most occupied *t*_2g_ orbital admixture) lies close to the phase boundary between ferromagnetic and A-type antiferromagnetic order, contributing to the magnetic fluctuations that suppress ferromagnetic order in YTiO_3_ (ref. ^11^). Therefore, depending on the excited phonon, the orbital state may be either driven closer to or farther from the phase boundary and the associated fluctuations, thereby weakening or improving ferromagnetism, respectively (Fig. 4c). DFT calculations of the orbital gap as a function of phonon displacement for the 4, 9 and 17 THz modes corroborate this hypothesis, showing a change in orbital polarization and ferromagnetic-favouring superexchange consistent with experimental findings (Fig. 4b). Because these effects (modulation of the orbital wavefunction and gap) are predicted to be quadratic in the amplitude of the optically driven phonons, oscillatory atomic displacements are converted into an average rectified change of the orbital properties. Ultimately, this scenario would lead to an effective pump-induced modification of the magnetic exchange or spin stiffness, as reflected in the MOKE signal.

**Extended Data Fig. 1:**
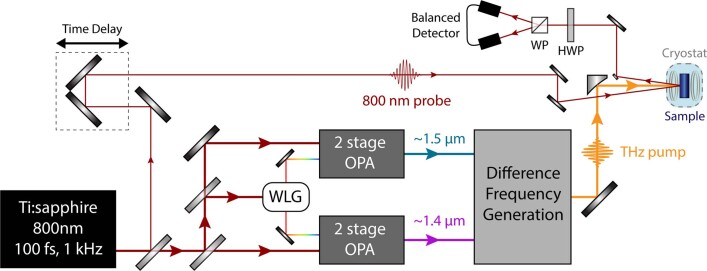
Schematic of the experimental THz pump - MOKE probe setup. OPA = optical parametric amplifier, HWP = half-waveplate, WP = Wollaston prism.

With these considerations in mind, we next discuss the timescales observed in our experiment. As shown in Extended Data Fig. 8, the transition from the equilibrium to the photo-induced ferromagnetic state occurs within roughly 10–40 ps, depending on the phonon being pumped. Once achieved, this non-equilibrium state seems to be metastable, requiring many nanoseconds before returning to equilibrium. The rapid change of magnetic order at early times is surprising because of the inefficient transfer of angular momentum expected for a magnetic insulator such as YTiO_3_ with relatively weak anisotropy and atomic spin–orbit coupling^34,38^. Hence, to explain the fast rise time, an extra interaction channel needs to be activated. As shown in Extended Data Fig. 8b, the rise time closely matches the coherent lifetime of the driven phonon, which is excited within the duration of the pump pulse (Extended Data Fig. 1) but rings for a time determined by its spectral linewidth. A simplified spin–orbital model shows that modulation of the crystal field arising from the large amplitude phonon oscillations accelerates the spin-flip relaxation rate relative to the equilibrium spin–orbit pathway, as schematically shown in Supplementary Fig. 4. We expect this dynamical channel to close once the driven phonons have decayed, trapping the system in a long-lasting, metastable state^39^ (see Supplementary Information for a more detailed discussion). The rigidity of the non-equilibrium magnetization is consistent with a strong bottleneck for magnetic relaxation in the absence of coherent phonons, leading to the long time scale associated with the return to equilibrium.

The mechanisms discussed above should be contrasted with the picture discussed in ref. ^34^, in which phonon excitation in yttrium iron garnet leads to exchange modulation by means of enhanced stochastic ligand motion, reducing the overall magnetization. In that case, the modulation sets in after the lattice system has thermalized, whereas here, in YTiO_3_, the change in ferromagnetic ordering is induced during the coherent lifetime of the phonon modes and starting well before complete lattice thermalization. We can also rule out temperature-induced effects from the pump, as the magnetization increases for some pumped modes, whereas heating would always decrease the magnetization in equilibrium. The induced magnetization change is found to switch sign depending on the phonon being pumped, indicating that the nature of the coherent structural deformations is important, rather than solely the total energy deposited into the lattice. Another possibility may involve coupling of driven phonons to strain; however, this scenario fails to explain important aspects of the experiment, such as the phonon-dependent sign change and the mechanism of angular momentum transfer. In principle, dynamical effects not considered so far, including nonlinear phonon couplings^35,36^ and non-equilibrium quasiparticle distributions may also play a role, and further experiments and theory are desirable to fully understand the microscopic origin of the high-temperature photo-induced ferromagnetism.

## Conclusion

To conclude, the experiments reported here underscore the power of resonant lattice excitation to enhance magnetic phases by exploiting their strong coupling to crystal structure^29–33^ and demonstrate a pathway to bring non-equilibrium functionalities to higher temperatures. The general approach is relevant not just to magnetism in titanates, but to the wider group of quantum materials which show unwanted degeneracies and fluctuating orders^40–42^. Finally, we emphasize that our results represent a rare case of light-driven symmetry breaking and can be viewed in the same broad class of discoveries as optically enhanced ferroelectricity, superconductivity or charge orders^43–45^.

## Methods

### Optical set-up and MOKE detection

**Extended Data Fig. 2:**
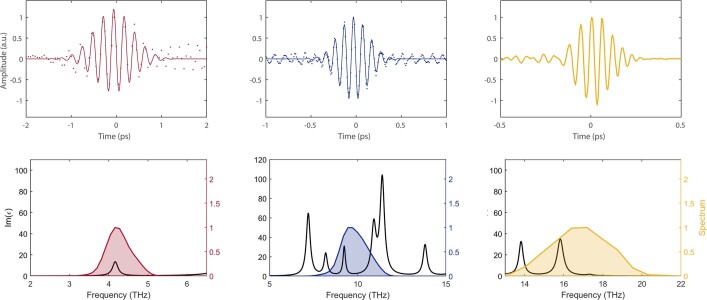
Characterization of the THz excitation pulses. Representative electro-optic sampling traces (top) and associated spectra (bottom) for the three different THz excitation pulses used in the experiment. The pulse durations are approximately 360 fs at 4 THz (left), 205 fs at 9 THz (center), and 160 fs at 17 THz (right). Solid lines are Gaussian pulse fits used to extract nominal pulse durations. Spectra shown in the bottom panels are obtained from fast Fourier transformation of the raw time-domain data.

Our MOKE measurements were carried out using the experimental set-up shown in Extended Data Fig. 1. The THz pump pulses were created by the chirped pulse difference frequency generation scheme described in detail in refs. ^31,46^. A Ti:sapphire regenerative amplifier (100 fs pulse length, 800 nm centre wavelength, 1 kHz repetition rate) fed two independently tuneable optical parametric amplifiers (OPAs) seeded by a common white light continuum, whose signal outputs produced roughly 70 fs long near-infrared pulses with centre wavelengths between 1,250 and 1,550 nm. A linear chirp was imparted on the OPA outputs by sending them through two transmission grating pairs, after which difference frequency mixing in a roughly 400 μm thick crystal of DAST (4-*N*,*N*-dimethylamino-4′-*N*′-methyl-stilbazolium tosylate) produced the desired THz transient. The centre frequency and bandwidth of the THz pulses were modified by choosing the wavelengths and the chirps of the near-infrared OPA outputs, respectively. Examples of the THz electric field waveforms and their associated spectra are shown in Extended Data Fig. 2.

The generated THz pulses were focused onto a YTiO_3_ single crystal mounted in a liquid helium cryostat equipped with a 5 T superconducting magnet. The THz propagation direction and the external magnetic field were both oriented normal to the (001) surface of the sample (that is, parallel to the ferromagnetic *c* axis). The THz electric field was linearly polarized parallel to the *b* axis.

**Extended Data Fig. 3:**
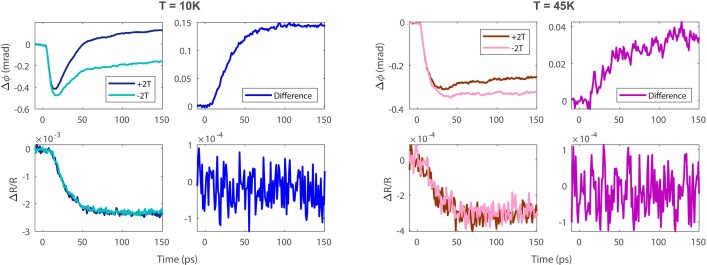
Raw time-resolved MOKE and reflectivity data. Comparison between the measured MOKE angles (upper plots) and the transient reflectance (lower plots) with 9 THz pumping for two representative temperatures above and below Tc, showing that while the time-resolved MOKE signal has different dynamics for positive and negative magnetic fields, the integrated probe reflectivity does not.

A small portion of the 800 nm amplifier output was used for the MOKE detection. These probe pulses were time delayed and focused onto the sample at a small angle (roughly 5°) relative to the sample normal in a polar MOKE geometry. The incident probe was *s*-polarized perpendicular to the THz polarization to eliminate artefacts from field-driven birefringence. The rotation of the polarization axis (*ϕ*) of the reflected probe pulses was determined using a standard balanced detection system, consisting of a half-wave plate (HWP), a Wollaston prism and a balanced photodiode. Before each pump-probe measurement, the HWP was adjusted to set the balanced photodiode output to zero for every temperature and magnetic field. The pump-induced polarization rotation changes (Δ*ϕ*) and transient reflectance (Δ*R*) were measured simultaneously from the difference and sum channels of the balanced photodiode, allowing us to rule out isotropic, non-magnetic contributions to the MOKE signal (Extended Data Fig. 3 and Methods section Jones matrix analysis). The time-resolved changes to the MOKE angle following pump excitation, which are shown in the main text, are defined as

1 $$\varDelta {\phi }_{{\rm{M}}}(t)=\frac{\varDelta \phi (+H,t)-\varDelta \phi (-H,t)}{2},$$

where *H* is the external magnetic field.

### Sample preparation and equilibrium measurements

The high-quality, stoichiometric YTiO_3_ single crystals were grown by a crucible-free floating zone method in Ar/H_2_ = 50/50 flow. A four-mirror type image furnace (CSI) equipped with 1.5 kW halogen lamps was used. The stoichiometry and structure, as well as the thermodynamic, magnetic and optical properties of the sample, have been previously characterized using energy-dispersive X-ray analysis, powder and single crystal X-ray diffraction, thermal gravimetry and/or differential thermal analysis, SQUID magnetometry and spectroscopic ellipsometry. In addition to the information provided here, a detailed description of the sample preparation and characterization can be found in ref. ^17^.

### MOKE signal calibration

**Extended Data Fig. 4:**
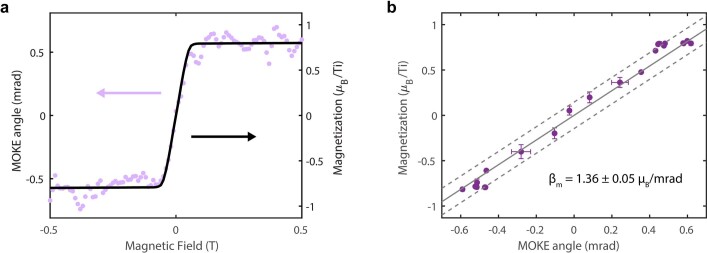
Determining the magneto-optical coefficient from static MOKE measurements. (a) MOKE angle as a function of magnetic field in the absence of a pump (purple dots, left axis) together with the equilibrium magnetization from VSM (black line, right axis), both measured at 10 K. (b) Correlation plot of the MOKE angle vs. magnetization, showing a linear dependence over the measured range. Binned data from (a) are shown as purple dots. The solid and dashed lines represent the linear fit and the 1σ prediction bounds.

To determine the absolute magnetization of YTiO_3_ in the pump-induced state, as reported in Figs. 3b and 4a, we calibrated the MOKE angle on the basis of equilibrium measurements. Without the THz pump impinging on the sample, we measured the static MOKE angle, *ϕ*_M_, as a function of external magnetic field, *H*. The signal was corrected for a linear background that arises because of the diamagnetic response of the cryostat windows. The resulting static MOKE measurement is shown in Extended Data Fig. 4a), providing the dependence *ϕ*_M_(*H*). Separately, on the same YTiO_3_ single crystal, we carried out measurements of the magnetization as a function of magnetic field, *M*(*H*), using a vibrating sample magnetometer (Quantum Design). By correlating the two measurements, we are able to obtain the calibration curve relating the MOKE angle to the magnetization (Extended Data Fig. 4b, which is linear over the meaured field range: $M={\beta }_{{\rm{M}}}{{\phi }}_{{\rm{M}}}$. The magneto-optical coefficient determined from a fit to the calibration curve is ${\beta }_{{\rm{M}}}\,{\rm{=\; 1.36}}\pm 0.05$ *μ*_B_ mrad^−1^.

This analysis is applied to the time-resolved MOKE data to obtain the non-equilibrium magnetization in the pump-induced state (${M}_{{\rm{pumped}}}$) by extrapolating the linear dependence. For a given field,

2 $${M}_{{\rm{pumped}}}(H,t)={\beta }_{{\rm{M}}}({{\phi }}_{{\rm{M}}}(H)+\varDelta {{\phi }}_{{\rm{M}}}(t)),$$

where Δ*ϕ*_M_(*t*) is the pump-induced change in the MOKE angle. As noted in the main text, a positive Δ*ϕ*_M_ corresponds to an increase in *M* with respect to the equilibrium ferromagnetic magnetization, while a negative Δ*ϕ*_M_ corresponds to a reduction in *M*.

**Extended Data Fig. 5:**
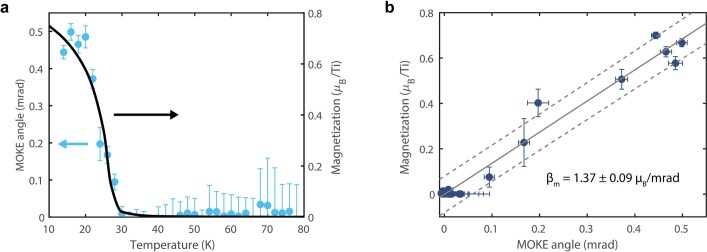
Temperature-dependent magneto-optical properties of YTiO_3_. (a) Static MOKE angle as a function of temperature (blue points, left axis) together with the equilibrium magnetization measured by VSM (black line, right axis) with H = 0.05 T. (b) Correlation plot of the MOKE angle vs. magnetization over this temperature range, showing that the magneto-optical coefficient remains constant within experimental error. The solid and dashed lines represent the linear fit and the 2σ prediction bounds.

The calibration procedure is repeated for each temperature, and it is found that *β*_M_ remains constant within the experimental error for temperatures below *T*_c_ = 27 K, as shown in Extended Data Fig. 5, and agrees with the value extracted from Extended Data Fig. 4. We note that the observed temperature independence of *β*_M_ agrees with magneto-optical studies carried out around the critical region in other ferromagnetic compounds^47^. We take the value of ${\beta }_{{\rm{M}}}\,{\rm{=\; 1.37}}\pm 0.09$ *μ*_B_ mrad^−1^ determined from the temperature dependence to obtain the non-equilibrium magnetization above *T*_c_.

The experimental values for the total magnetization in the pump-induced state (reported in Figs. 3b and 4a) are obtained from equation (2), where the term ${\beta }_{{\rm{M}}}{\phi }_{{\rm{M}}}(H)$ is replaced by the equilibrium *M*(*H*) determined by vibrating sample magnetometer, which has a relative uncertainty of less than 1% and does not contribute significantly to the uncertainty in ${M}_{{\rm{pumped}}}$. The error bars on these figures are given by two main contributions: the uncertainty in the determination of *β*_M_ (described above) and the uncertainty in the maximum value of Δ*ϕ*_M_ at long times. Due to the slow decay, the maximum saturated value of Δ*ϕ*_M_ was determined by averaging the signal between *t* = 100 and 200 ps, with the uncertainty given by the standard error of those data points. The maximum value of Δ*ϕ*_M_ can also be determined from the fits to the data (as in the Methods section Time scales of pump-induced magnetization). The extracted values and error bars from the two approaches were found to be equivalent.

### Non-magnetic contributions to MOKE signal

Key to our interpretation is the fact that any non-magnetic pump-induced changes to the optical properties of the sample negligibly affect our experimental measurements. To validate this claim, we present here a full analysis of the various contributions to our experimental signal formulated using Jones calculus and argue that we are only sensitive to changes in the magnetization.

### Jones matrix analysis

As described in the section Optical setup and MOKE detection, we use a standard balanced detection scheme in which the polarization after the sample is rotated by a HWP and split into orthogonal linear components labelled *s* and *p*, which are aligned with the horizontal and vertical axes in the laboratory frame. The intensities of these components *I*_*p*_ and *I*_*s*_ comprise the experimental polarization rotation signal:

3 $${\phi }^{{\rm{sig}}}=\frac{1}{2}\frac{\varDelta I}{{I}_{{\rm{sum}}}},$$

where $$\varDelta I={I}_{s}-{I}_{p}$$ and $${I}_{{\rm{sum}}}={I}_{s}+{I}_{p}$$.

The general polarization in the *s* and *p* basis can be described by the general Jones vector,

4 $${\bf{E}}=[\begin{array}{c}{E}_{p}\\ {E}_{s}\end{array}]$$

The Jones matrix for the HWP, whose primary axis is set at an angle *θ* with respect to the vertical is,

5 $$P(\theta )=[\begin{array}{cc}-{\rm{\cos }}2\theta  & {\rm{\sin }}2\theta \\ {\rm{sin}}2\theta  & {\rm{\cos }}2\theta \end{array}]{\rm{}}.$$

After the sample, the beam is routed by two metallic mirrors with an angle of incidence (AOI) of 5°. For a general metallic mirror, the Jones matrix can be written as,

6 $${J}_{{\rm{MIR}}}={r}_{{\rm{m}}}{{\rm{e}}}^{{{i\kappa }}_{{\rm{p}}}}[\begin{array}{cc}1 & 0\\ 0 & A{{\rm{e}}}^{i\varDelta \kappa }\end{array}]{\rm{}}.$$

In the ideal case, the reflectivity terms *r*_m_ and *A* = 1 and the phase shifts ${\kappa }_{{\rm{p}}}{\rm{=\; 0}}$ and $\varDelta \kappa =\pi$. For the silver mirrors used in our experiment, *r*_m_ ≅ 0.981, *A* = 0.995, ${\kappa }_{{\rm{p}}}\,{\rm{=\; 0.110}}\pi$ and $\varDelta \kappa =-1.001\pi$.

Following ref. ^48^, the YTiO_3_ sample is represented by the Fresnel reflection matrix

7 $$S=[\begin{array}{cc}{r}_{pp} & {r}_{ps}\\ {r}_{sp} & {r}_{ss}\end{array}],$$

with ${r}_{ps}=-{r}_{sp}$. At normal incidence, ${r}_{ss}={r}_{pp}=r=\frac{n+1}{n-1}\,=\,0.395$, and the MOKE rotation angle

8 $$\phi =\frac{{r}_{ps}}{{r}_{ss}}=\frac{n\gamma M}{{n}^{2}-1}={\alpha }_{{\rm{M}}}\,M{\rm{}}.$$

The refractive index of YTiO_3_ at the probe wavelength, *n* = 2.304, is taken from ref. ^17^. The coefficient *γ* is the magneto-optical constant. The terms multiplying *M* are combined into a generalized magneto-optical coefficient *α*_M_. In the section Potential magneto-optical effects, we explain how changes in *ϕ* induced by the pump can be related to changes in the magnetization Δ*M* through the coefficient *α*_M_. In this section, we describe how the signal that we observe in the real experimental set-up does indeed provide an accurate measure of the intrinsic MOKE angle *ϕ*.

With a finite AOI of *α* = 5°, the reflection matrix becomes slightly anisotropic,

9 $$S={r}_{ss}[\begin{array}{cc}-\eta  & {\phi }^{{\prime} }\\ -\eta {\phi }^{{\prime} } & 1\end{array}]\mathrm{}.$$

where ${r}_{ss}=\frac{n{\rm{\cos }}{\alpha }_{n}-{\rm{\cos }}\alpha }{n{\rm{\cos }}{\alpha }_{n}+{\rm{\cos }}\alpha }\,=\,{\rm{0.396}}$ and $\eta =|\frac{{r}_{pp}}{{r}_{ss}}|\,=\,0.993$ describes the anisotropy of the Fresnel reflectivities. The internal angle from Snell’s law is ${\alpha }_{n}={{\rm{\sin }}}^{-1}\left(\frac{1}{n}{\rm{\sin }}\alpha \right)\approx {2.2}^{^\circ }$. The apparent rotation angle ${\phi }^{{\prime} }$ is slightly modified from the pure MOKE angle *ϕ* of equation (8) due to the finite AOI: ${\phi }^{{\prime} }=c\phi$, where $c=\frac{{\rm{\cos }}\alpha }{{\rm{\cos }}(\alpha -{\alpha }_{n})}$. Hence, whereas the finite AOI does slightly modify the reflection parameters, the deviations from the normal incidence case are small.

To determine the polarization measured from our balanced detection set-up, we can propagate the incident polarization through the Jones matrix of each element. If the incident polarization is perfectly *s*-polarized,

10 $${E}_{{\rm{in}}}=[\begin{array}{c}0\\ 1\end{array}],$$

then:

11 $${E}_{{\rm{out}}}=P(\theta )\cdot {J}_{{\rm{MIR}}}\cdot {J}_{{\rm{MIR}}}\cdot S\cdot {E}_{{\rm{in}}}=\frac{{r}_{ss}}{\sqrt{2}}{r}_{{\rm{m}}}^{2}{{\rm{e}}}^{2i{\kappa }_{{\rm{p}}}}[\begin{array}{c}{A}^{2}{{\rm{e}}}^{2i\varDelta \kappa }{\rm{\sin }}2\theta -{\phi }^{{\prime} }{\rm{\cos }}2\theta \\ {A}^{2}{{\rm{e}}}^{2i\varDelta \kappa }{\rm{\cos }}2\theta +{\phi }^{{\prime} }{\rm{\sin }}2\theta \end{array}]{\rm{}}.$$

For measurements of the pump-induced MOKE angle $\varDelta {\phi }_{{\rm{M}}}^{{\rm{sig}}}(H,t)$, we first apply the external field *H*, then select the HWP angle *θ* to compensate for the static field-induced rotation ${\phi }_{0}^{{\rm{sig}}}(H)$. This is accomplished by finding the angle ${\theta }_{{\rm{bal}}}$ for which the difference signal Δ*I* is zero, or equivalently, when the two outgoing polarization components *E*_*p*_ and *E*_*s*_ are equal. Here, the balancing condition is given by,

12 $${\theta }_{{\rm{bal}}}=\frac{1}{4}{{\rm{\tan }}}^{-1}\left(\frac{-2{A}^{2}{\phi }_{0}^{{\prime} }(H){\rm{\cos }}2\varDelta \kappa }{{A}^{4}+{\phi }_{0}^{{\prime} }{(H)}^{2}}\right)\approx \frac{\pi }{8}+1.007\frac{{\phi }_{0}(H)}{2}.$$

Then, fixing the HWP setting at this angle and allowing for pump-induced changes in the magnetization, which would yield a time-dependent change in the intrinsic MOKE angle, $\varDelta {\phi }_{{\rm{M}}}(H,t)=\varDelta ({\alpha }_{{\rm{M}}}\,M)$, the resulting pump-induced signal is

13 $$\varDelta {\phi }_{{\rm{M}}}^{{\rm{sig}}}(H,t)\approx \frac{{\rm{c}}{\rm{o}}{\rm{s}}2\varDelta \kappa }{{A}^{2}}\varDelta {\phi }_{{\rm{M}}}^{{\prime} }(H,t){\rm{=\; 1.007}}\varDelta {\phi }_{{\rm{M}}}(H,t){\rm{}}.$$

The approximation holds for small angles *ϕ*_0_ and Δ*ϕ*_M_, which is certainly satisfied in our experiment, where the maximum we observe is less than 1 mrad or 0.06°. Hence, from equation (12), with a perfectly *s*-polarized incident beam, the detected time-resolved changes in the MOKE angle are proportional to the intrinsic time-resolved MOKE angle coming from pump-induced changes to the magnetization of YTiO_3_ with a mismatch of less than 1%. Notice, importantly, that as the difference of the two polarization states is normalized by their sum in our signal, the reflectivity and (non-magnetic) pump-induced changes thereof drop out of the expression for the detected signal and do not affect it at all under these ‘ideal’ experimental conditions. The only errors result from the imperfections of the mirrors used after the sample.

We can also investigate additional errors that might result from a slight misalignment of the incident polarization from perfect *s* polarization. We can imagine that the incident polarization is rotated from the ideal *s* polarization by a small angle *δ*,

14 $${E}_{{\rm{in}}}=[\begin{array}{c}{\rm{\sin }}\delta \\ {\rm{\cos }}\delta \end{array}]{\rm{}}.$$

Following the same procedure as above, we get for the static case,

15 $$\begin{array}{l}{E}_{{\rm{out}}}={r}_{ss}{r}_{{\rm{m}}}^{2}{{\rm{e}}}^{2i{\kappa }_{{\rm{p}}}}\\ \,[\begin{array}{l}{A}^{2}{{\rm{e}}}^{2i\varDelta \kappa }{\rm{\sin }}2\theta ({\rm{\cos }}\delta -\eta {\phi }^{{\prime} }{\rm{\sin }}\delta )-{\rm{\cos }}2\theta ({\phi }^{{\prime} }{\rm{\cos }}\delta -\eta {\rm{\sin }}\delta )\\ {A}^{2}{{\rm{e}}}^{2i\varDelta \kappa }{\rm{\cos }}2\theta ({\rm{\cos }}\delta -\eta {\phi }^{{\prime} }{\rm{\sin }}\delta )+{\rm{\sin }}2\theta ({\phi }^{{\prime} }{\rm{\cos }}\delta -\eta {\rm{\sin }}\delta )\end{array}].\end{array}$$

**Extended Data Fig. 6:**
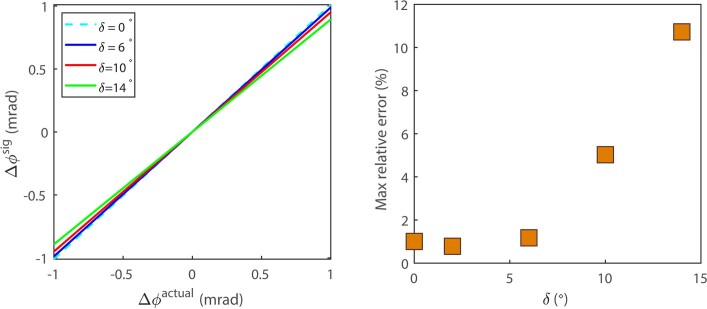
Analysis of potential sources of error in MOKE measurement. (left) The detected pump-induced MOKE signal $\varDelta {\varphi }^{{sig}}$ vs. the actual pump-induced change in MOKE angle for different incident probe misalignments, δ. (right) The maximum relative deviation $\left|\frac{\varDelta {\varphi }^{{sig}}-\varDelta {\varphi }^{{actual}}}{\varDelta {\varphi }^{{actual}}}\right|$ over the range −1 to 1 mrad for δ = 5°.

The algebraic forms of the subsequent equations for the ${\theta }_{{\rm{bal}}}$ and $\varDelta {\phi }_{{\rm{M}}}^{{\rm{sig}}}$ are cumbersome, so instead we numerically analyse the extracted signal. Fixing ${\phi }_{0}(H)=1$ mrad (twice the value measured statically at 2 T) to provide an upper bound, we compute the resulting pump-induced MOKE signal $\varDelta {\phi }_{{\rm{M}}}^{{\rm{sig}}}$ for varying *δ* and compare to the actual intrinsic value of Δ*ϕ*_M_ from the sample (Extended Data Fig. 6).

In all cases, $\varDelta {\phi }_{{\rm{M}}}^{{\rm{sig}}}$ is linearly proportional to Δ*ϕ* with small deviations only visible for large *δ*. It is unlikely that a misalignment of more than a few degrees in the probe polarization would appear in our experiment; for realistic values of *δ* *<* 5°, the error between the measured and actual value of Δ*ϕ*_M_ is 1% or less.

We note that, even in this geometry with a misaligned probe polarization, any pump-induced changes in the overall sample reflectivity (here, given by Δ*r*_*ss*_) are again cancelled out due to the signal normalization. However, pump-induced changes to the anisotropic Fresnel factors *η* and *c* could still influence the signal. These effects turn out to be extremely small and well within the uncertainty in the experimental measurements. To see this, one can determine the pump-induced change in the refractive index from the measured change in the reflectivity Δ*r*/*r* and the Fresnel equations,

16 $$\varDelta n\approx \left(\frac{\varDelta r}{r}\right)\cdot {\left(\frac{\partial {\rm{ln}}r}{\partial n}\right)}^{-1}=\left(\frac{\varDelta r}{r}\right)\cdot \left(\frac{({n}^{2}-1)\sqrt{{\rm{\cos }}2\alpha +2{n}^{2}-1}}{2\sqrt{2}n{\rm{\cos }}\alpha }\right)$$

From Extended Data Fig. 3, we see that the magnitude of Δ*r*/*r* is on the order of 3 × 10^−3^ at its largest. Plugging in for *n* and *α* from above, we can get an upper bound of $|\varDelta n|\approx 0.006$. The resulting pump-induced changes in *η* and *c* would then be,

17 $$\varDelta \eta \approx \frac{\partial \eta }{\partial n}\varDelta n\approx -2\times {10}^{-5}$$

18 $$\varDelta c\approx \frac{\partial c}{\partial n}\varDelta n\approx -5\times {10}^{-6}\mathrm{}.$$

With these deviations, the total error in our measured MOKE signal is only around 0.77% and the contribution specifically from Δ*η* and Δ*c* is extremely small: on the order of 0.001%. Therefore, after a thorough analysis and estimate of the errors of our experimental set-up, we conclude that non-magnetic effects arising from imperfect optics and pump-induced changes in reflectivity do not affect our experimental signal.

### Potential magneto-optical effects

On the basis of the Jones matrix discussion above, we established that our measured signal describes the intrinsic MOKE angle of the sample. One additional assumption in our analysis is that pump-induced changes in *β*_M_ (that is, the magneto-optical constant) are negligible. To verify this assumption, we consider the different contributions to the pump-induced change of the MOKE angle^49,50^,

19a $$\varDelta {\phi }_{{\rm{M}}}\,=\,\varDelta ({\alpha }_{{\rm{M}}}\,M)$$

19b $$\,=\,M\varDelta {\alpha }_{{\rm{M}}}+{\alpha }_{{\rm{M}}}\,\varDelta M,$$

where ${\alpha }_{{\rm{M}}}={\beta }_{{\rm{M}}}^{-1}$ is the magneto-optical coefficient from equation (8). Equation (19b) can be written in terms of the relative changes ($\delta {\phi }_{{\rm{M}}}=\frac{\varDelta {\phi }_{{\rm{M}}}}{{\phi }_{{\rm{M}}}}$, $\delta {\alpha }_{{\rm{M}}}=\frac{\varDelta {\alpha }_{{\rm{M}}}}{{\alpha }_{{\rm{M}}}}$ and $\delta M=\frac{\varDelta M}{M}$), as

20 $$\delta {\phi }_{{\rm{M}}}=\delta {\alpha }_{{\rm{M}}}+\delta M{\rm{}}.$$

Both *ϕ*_M_ and *α*_M_ are complex quantities: ${\phi }_{{\rm{M}}}^{{\prime} }={\rm{Re}}({\phi }_{{\rm{M}}})$ is the MOKE rotation and ${\phi }_{{\rm{M}}}^{{\prime\prime} }={\rm{I}}{\rm{m}}({\phi }_{{\rm{M}}})$ is the MOKE ellipticity. Assuming the dynamics associated with the real and imaginary parts of *α*_M_ follow each other, $\delta {\alpha }_{{\rm{M}}}^{{\prime} }=k\delta {\alpha }_{{\rm{M}}}^{{\prime\prime} }$, one can isolate the relative change in the magnetization^50^,

21 $$\delta M=\frac{\delta {\phi }_{{\rm{M}}}^{{\prime} }-k\delta {\phi }_{{\rm{M}}}^{{\prime\prime} }}{1-k}{\rm{}}.$$

**Extended Data Fig. 7:**
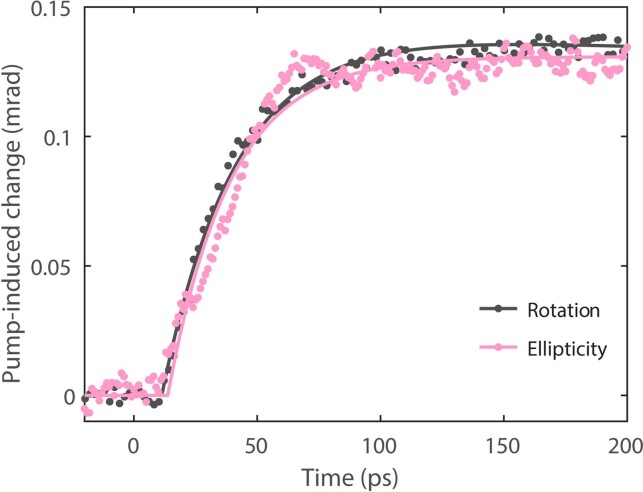
Dynamics of pump-induced MOKE rotation and ellipticity signals. Comparison of the pump-induced MOKE rotation (grey) and ellipticity (pink) angles. The two curves overlap, suggesting that the signal represents pure magnetization dynamics, rather than pump-induced changes in the magneto-optical coefficient. These measurements were taken with 9 THz pump excitation at T = 10 K, and H = ±2 T, but all other pump wavelengths, temperatures, and fields show similar behavior.

In Extended Data Fig. 7, we compare the pump-induced MOKE rotation to the ellipticity, measured by replacing the HWP in the detection with a quarter-wave plate. We find that the two signals lie on top of each other within our experimental error, indicating that $\delta {\phi }_{{\rm{M}}}^{{\prime} }\approx \delta {\phi }_{{\rm{M}}}^{{\prime\prime} }$. Then, equation (21) simplifies to $\delta M\approx \delta {\phi }_{{\rm{M}}}^{{\prime} }$, or, expanding,

22 $$\varDelta {\phi }_{{\rm{M}}}\approx {\alpha }_{{\rm{M}}}\,\varDelta M{\rm{}}.$$

That is, the dynamics we observe can be attributed to true magnetization dynamics.

In addition, no probing volume correction is needed because the penetration depth at the probe wavelength (${\delta }_{{\rm{probe}},800{\rm{nm}}}$ = 178 nm) is much shorter than those at all of the pump wavelengths used in the experiment (${\delta }_{{\rm{pump}},4{\rm{THz}}}$ = 3.5 μm, ${\delta }_{{\rm{pump}},9{\rm{THz}}}$ = 6.6 μm, ${\delta }_{{\rm{pump}},17{\rm{THz}}}$ = 600 nm). The penetration depths were extracted from our measured infrared spectra (Fig. 2a and ref. ^51^).

### Time scales of pump-induced magnetization

**Extended Data Fig. 8:**
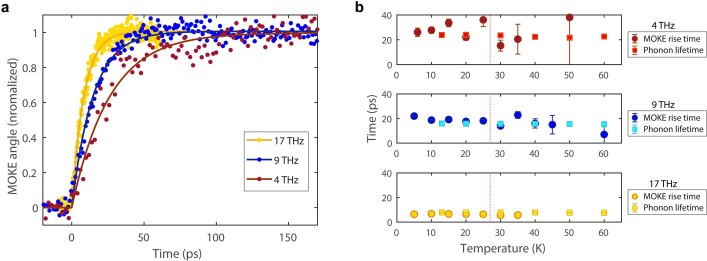
Non-equilibrium magnetization dynamics and phonon lifetimes. (a) Normalized time-resolved MOKE signals for different phonon excitations at T = 6 K. (b) Fitted rise times of the pump-induced MOKE signals (circles) and the equilibrium phonon lifetimes (squares) as a function of temperature. The rise times are extracted from the fits of the data to Eq. 23. The phonon lifetimes are determined from the inverse linewidth of the modes in infrared vibrational spectra taken at different temperatures (Extended Data Fig. 10).

**Extended Data Fig. 9:**
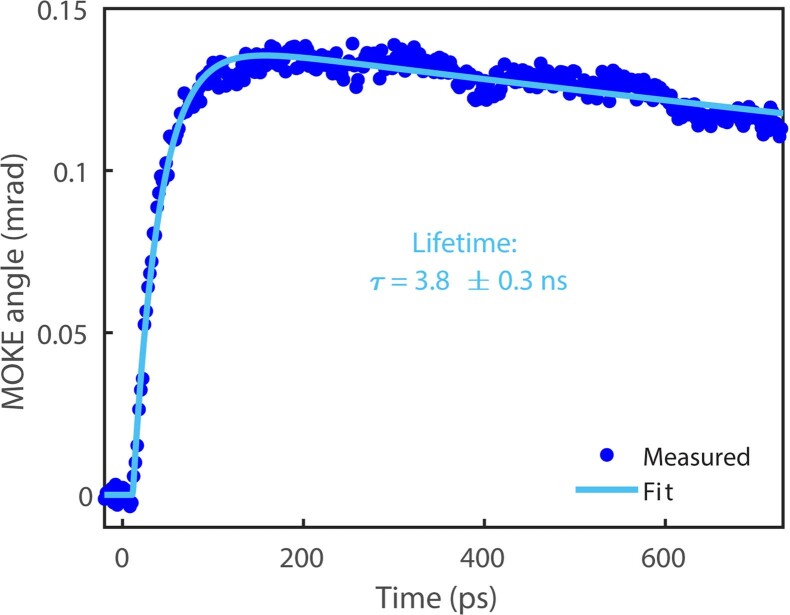
Time scale of pump-induced magnetization decay. Measurement of the pump-induced MOKE angle with longer time delay. The measurements were taken with 9 THz pump excitation at T = 10 K, and H = ±2 T. The solid line shows the fit to the data using Eq. 23.

The time-resolved data presented in the main text focus on the early to intermediate time response of the pump-induced state. On the time scales of those measurements (Δ*t* < 200 ps), the signal grows following pump excitation then remains relatively constant, allowing one to analyse the saturated magnetic behaviour. The saturation of the pump-induced response over hundreds of picoseconds points to the existence of a metastable non-equilibrium magnetic state, which persists for much longer than the coherent structural response induced by the resonant phonon excitation (typically tens of picoseconds, Extended Data Fig. 8). To figure out the lifetime of the metastable state, we also carried out time-resolved MOKE measurements over longer time scales, up to roughly 1 ns. A representative measurement taken with 9 THz pump excitation at low temperature (*T* = 10 K) is shown in Extended Data Fig. 9. We observe a sharp rise and slow decay of the pump-induced MOKE angle Δ*ϕ*_M_, which can be fit by a decaying exponential model:

23 $$\varDelta {\phi }_{{\rm{M}}}(t)=A(1-{{\rm{e}}}^{-t/\sigma }){{\rm{e}}}^{-t/\tau }{\rm{}}.$$

The time constants *σ* and *τ* represent the rise time to reach saturation and the lifetime of the non-equilibrium state, respectively, and *A* is the saturation value of the MOKE angle. We obtain fitted values of *σ* = 30 ± 2 ps and *τ* = 3.8 ± 0.3 ns. The several-nanosecond lifetime demonstrates the metastability of the pump-induced phase, as this time scale is much longer than any external time scale of the system.

To help us understand the mechanism leading to the formation of the non-equilibrium magnetic state, we compare the MOKE signal rise time to the lifetime of the driven phonon as a function of temperature (Extended Data Fig. 8). The time scales vary on the basis of the mode being excited. At low temperatures, the rise time is roughly 30 ps for the 4 THz mode, 20 ps for the 9 THz mode and 10 ps for the 17 THz mode. The lifetimes of the phonons at *T* = 13 K, determined from equilibrium vibrational spectra, are 24, 16 and 8, respectively, remaining relatively constant crossing through *T*_c_. These values provide a lower bound assuming the spectral features are homogeneously broadened; if inhomogeneous broadening plays a role, the decoherence time of the driven phonon could be longer. From these comparisons, we conclude that the rise time to reach the saturated, long-lived magnetization state is roughly equivalent to the lifetime of the driven phonon for all pump excitations studied. The fact that the lattice is in a coherently driven state throughout most of the transition points to a non-thermal, phonon-mediated mechanism underlying the dynamics of the pump-induced magnetization. A detailed discussion of the proposed mechanism, which is based on the coupling between coherently driven phonons, the orbital state, and the associated magnetic order, is presented in the Supplementary Information.

### Infrared spectra and experimental spin–phonon couplings

We used synchrotron-based spectroscopic ellipsometry to accurately determine the infrared phonon spectra of YTiO_3_, as described in ref. ^51^. The ellipsometric measurements in the frequency range from 9 to 85 meV (70 to 690 cm^−1^) used synchrotron edge radiation of the 2.5 GeV electron storage ring at the IR1 beamline of the Karlsruhe Research Accelerator at the Karlsruhe Institute of Technology, Germany, and were performed using a home-built ellipsometer in combination with a Bruker IFS 66v/S Fourier-transform infrared spectrometer. The ellipsometric parameters $\varPsi$ and *Δ*, measured at an AOI of 15°, define the complex ratio ${r}_{p}/{r}_{s}={\rm{\tan }}(\varPsi ){{\rm{e}}}^{i\varDelta }$, where *r*_*p*_ and *r*_*s*_ are the complex Fresnel coefficients for light polarized parallel and perpendicular to the plane of incidence, respectively. For anisotropic samples, a direct analytical inversion of the ellipsometric parameters into the diagonal components of the complex dielectric tensor *ε*_*xx*_, *ε*_*yy*_ and *ε*_*zz*_ is not possible, and a numerical regression procedure is required. To determine the dielectric function of YTiO_3_, we measured on the *ac* and *bc* surfaces cut from the same crystal, with *a* or *b* axes aligned either parallel or perpendicular to the plane of incidence, respectively. A nonlinear fitting procedure was applied to extract point by point the complex dielectric response throughout the covered spectral range. Figure 2a in the main text shows the true *b* axis complex dielectric response *ε* = *ε*_*yy*_ extracted from the raw ellipsometry spectra $\varPsi (\omega )$ and *Δ*(*ω*). The transverse optical phonon modes appear as peaks in Im(*ε*).

**Extended Data Fig. 10:**
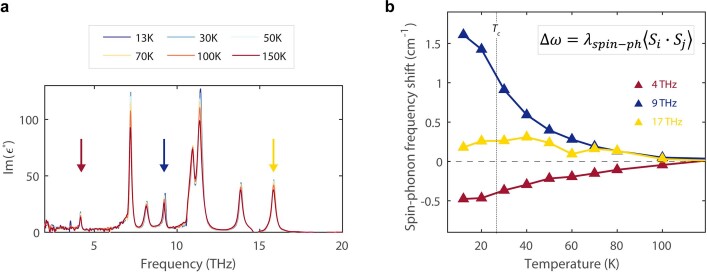
Equilibrium infrared spectroscopy and spin-phonon coupling in YTiO_3_. (a) Pseudo-dielectric function for the b-axis of YTiO_3_ plotted at several different temperatures. Arrows indicate the three modes excited in our experiment. (b) Frequency shifts for the 4, 9, and 17 THz modes as a function of temperature with the anharmonic background subtracted off. The additional shifts are related to spin-phonon coupling, and the anomalous behavior at high temperatures indicates the presence of local spin correlations in YTiO_3_ well above T_c_.

To study the changes in the parameters of the phonon modes with temperature, we used a simplified approach. The complex pseudo-dielectric function ε* in Extended Data Fig. 10a is derived by a direct inversion of $\varPsi$ and *Δ* measured on the *bc* plane with the *b* axis in the plane of incidence, assuming semi-infinite bulk isotropic behaviour of the crystal. Several features of the phonon modes are found to change with temperature, namely the amplitudes, linewidths and frequencies of the modes. Here, we focus on the frequency shifts with temperature. As YTiO_3_ is magnetic, there are two main contributions to the shift of the phonon frequency *Δω*, which can be written as

24 $$\frac{\varDelta \omega }{\omega }=\varDelta ({\rm{ln}}\omega )=\frac{\partial }{\partial T}({\rm{ln}}\omega )\varDelta T+\frac{\partial }{\partial (\langle {S}_{i}{S}_{j}\rangle )}({\rm{ln}}\omega )\varDelta (\langle {S}_{i}{S}_{j}\rangle ){\rm{}}.$$

where $\langle {S}_{i}{S}_{j}\rangle$ is the nearest-neighbour spin correlation function. The first term on the right-hand side is the contribution due to lattice anharmonicity. The second term arises due to spin–phonon coupling, which, for infrared-active modes, enters to the lowest order into the lattice potential as ${E}_{{\rm{spin-ph}}}=\omega \lambda \langle {S}_{i}{S}_{j}\rangle {Q}^{2}$, where *λ* is the spin–phonon coupling constant and *Q* is the phonon amplitude. The frequency shift associated with this term is $\varDelta {\omega }_{{\rm{spin-ph}}}\approx \lambda \langle {S}_{i}{S}_{j}\rangle$. To isolate the spin–phonon contribution, we subtract off the anharmonic background, which can be determined by fitting the data at high temperatures to equation (3.8) in ref. ^52^. Note that in reality, we use the frequency shift of the 7 THz phonon as the background function, as it can be described well by the anharmonic model throughout the entire temperature range, indicating negligible spin–phonon coupling for this mode. The spin–phonon frequency shift for the other modes then becomes $\varDelta {\omega }_{k,{\rm{spin-ph}}}(T)=\varDelta \omega (T)-{g}_{k}\varDelta {\omega }_{7{\rm{THz}}}$, where ${g}_{k}$ is determined by the condition $\varDelta {\omega }_{k,{\rm{spin-ph}}}\approx 0$ for *T* > 150 K, which is easily satisfied for all modes with $\mathrm{0.45 < }{g}_{k}\mathrm{ < 1.1}$.)

The temperature dependent spin–phonon frequency shifts for the three phonons driven in our pump-probe experiment are plotted in Extended Data Fig. 10b. At low temperatures, $\langle {S}_{i}{S}_{j}\rangle$ reaches a maximum so that the spin–phonon coupling constant *λ* is given by $\varDelta {\omega }_{{\rm{spin-ph}}}$. One can see from this analysis that the sign and magnitude of *λ* differ between the three modes. Furthermore, $\varDelta {\omega }_{{\rm{spin-ph}}}$ is non-zero all the way up to more than 100 K, indicating that $\langle {S}_{i}{S}_{j}\rangle$ is still finite at these temperatures well above *T*_c_. This high-temperature fluctuating spin order provides a potential basis for the non-equilibrium magnetic state we report in the main text.

## Online content

Any methods, additional references, Nature Portfolio reporting summaries, source data, extended data, supplementary information, acknowledgements, peer review information; details of author contributions and competing interests; and statements of data and code availability are available at 10.1038/s41586-023-05853-8.

## Supplementary information

Supplementary Information Additional discussion of DFT calculations (Supplementary Section 1) including Supplementary Figs. 1 and 2, details regarding magnetization dynamics (Supplementary Section 2) including Figs. 3–7, and additional references.

## Data Availability

Source data are provided with this paper. Further datasets collected for this study are available from the corresponding authors on reasonable request.

## Source data

Source Data Fig. 1
Source Data Fig. 2
Source Data Fig. 3
Source Data Fig. 4
