# Supplementary material for: Photo-induced high-temperature ferromagnetism in YTiO3
Source: Nature. 2023 May 3;617(7959):73–8. doi: 10.1038/s41586-023-05853-8 (PMC10156606; doi:10.1038/s41586-023-05853-8)
Supplement: Supplementary file 1 — Additional discussion of DFT calculations (Supplementary Section 1) including Supplementary Figs. 1 and 2, details regarding magnetization dynamics (Supplementary Section 2) including Figs. 3–7, and additional references. [file 41586_2023_5853_MOESM1_ESM.pdf]

nature portfolio

https://doi.org/10.1038/s41586-023-05853-8

Supplementary information

Photo-induced high-temperature ferromagnetism in $YTiO_3$

In the format provided by the authors and unedited

nature portfolio

https://doi.org/10.1038/s41586-023-05853-8

Supplementary information

Photo-induced high-temperature ferromagnetism in $YTiO_3$

In the format provided by the authors and unedited

Supplementary Information for "Photo-induced High Temperature Ferromagnetism in $YTiO_3$"

A. S. Disa[1,2], J. Curtis[3,4], M. Fechner[1], A. Liu[1], A. von Hoegen[1], M. Först[1], T.F. Nova[1], P. Narang[3,4], A. Maljuk[5], A.V. Boris[6], B. Keimer[6], and A. Cavalleri[1,7]

[1] *Max Planck Institute for the Structure and Dynamics of Matter, Hamburg, Germany*
[2] *School of Applied & Engineering Physics, Cornell University, Ithaca, USA*
[3] *John A. Paulson School of Engineering and Applied Sciences, Harvard University, Cambridge, USA*
[4] *College of Letters and Science, University of California, Los Angeles, USA*
[5] *Leibniz Institute for Solid State and Materials Research Dresden, Germany*
[6] *Max Planck Institute for Solid State Research, Stuttgart, Germany*
[7] *Clarendon Laboratory, Department of Physics, Oxford University, Oxford, UK*

Contents

S1 Density functional theory calculations **2**
S1.1 Equilibrium Properties 2
S1.2 Driven phonon amplitudes 3
S1.3 Spin-Phonon Coupling 3
S1.4 Wannier Functions 4
S1.5 Numerical Settings 4
S1.6 U-dependence 5

S2 Magnetization Dynamics **6**
S2.1 Equilibrium magnetization lifetime 6
S2.2 Driven magnetization lifetime 9

S1 Density functional theory calculations

To characterize the spin-lattice interaction in $YTiO_3$, we performed first-principles calculations in the framework of density functional theory (DFT). Our main aim here is to examine how the structural distortion due to the phonon excitation alters the magnetic exchange interactions. We computed first the DFT equilibrium structure, as well as the phonon spectrum and Ti magnetic exchange energies within this structure. Subsequently, we performed frozen phonon calculations, investigating how the magnetic interactions are modified by the displacement of specific eigenmodes. Lastly, to examine the effect of lattice distortions on the orbital polarization, we also calculated the localized t_{2g}-like Wannier functions for the phonon distorted structures. Wannierization of the t_{2g} Ti band was performed using projectors, including the maximal localization procedure [54].

The technical and numerical details of our calculation are outlined in Sec. S1.5.

S1.1 Equilibrium Properties

Investigating structural deformations upon displaced phonons requires a force-free reference structure. Hence, we first structurally relaxed the $YTiO_3$ unit cell to the lowest-energy state. We took the orthorhombic unit cell ($Pbnm$) with ferromagnetic spin order on the Ti atoms as a starting point. After relaxation, the computed lattice constants were $a = 5.34$ Å, $b = 5.68$ Å and $c = 7.61$ Å. In the final structure, the Y atoms occupy the $4c$-Wyckoff positions with $x = 0.02362$ and $y = 0.07503$. O atoms reside in two distinct positions: O(1) at $4c$ with $x = 0.63$ and $y = -0.05$, and O(2) at $8d$ with $x = 0.81$, $y = 0.19$ and $z = 0.56$. All values are in satisfactory agreement with the experimentally observed structure. For example, we compute $21.4°$ and $12.8°$ for the oxygen octahedral tilt and rotation angles in agreement with the experimental values of $20.1°$ and $12.5°$. The phonon spectrum of the optimized structure was computed using the Phonopy software package [55]. The relevant modes for this experiment are zone center phonons with polarization along the b-axis. The computed phonon frequencies are listed in Tab. S1.

Once the optimized $YTiO_3$ structure was obtained, we computed the magnetic exchange interactions. Following Ref. [56] we utilized a Heisenberg model with two magnetic exchange constants, J_{in} and J_{out}, describing the in-plane and out-of-plane Ti exchange interactions, respectively. J_{in} and J_{out} were computed by taking the difference between the total energy for distinct spin arrangements of the $YTiO_3$ unit cell. Specifically, we considered all four possible magnetic states of the $YTiO_3$ unit cell: ferromagnetic (FM), and A-type, C-type, and G-type antiferromagnetic (A-AFM, C-AFM, G-AFM). From these calculations, we find $J_{\text{in}} = -1.8$ meV and $J_{\text{out}} = -0.95$ meV, confirming the FM ground state.

Mode number	Frequency (THz)	$Z^*(e/\sqrt{\text{u}}\text{Å})$
1	4.1	0.26
2	7.0	0.89
3	8.0	0.32
4	9.0	0.33
5	10.7	0.83
6	11.6	1.44
7	14.0	0.61
8	15.9	1.36
9	16.7	0.74

Table S1: Computed phonon frequencies for B_{2u} symmetry (b-axis polarized) infrared-active phonon modes of $YTiO_3$, together with their mode effective charges. The phonons pumped in the experiment are highlighted in the same color as they are presented in main text.

We additionally examined if the (atomic) spin-orbit interaction is essential to the magnetism in $YTiO_3$ by including it explicitly in the DFT calculations. We first computed the magnetocrystalline anisotropy energy (MCA) by taking energy differences for the three distinct FM spin alignments, oriented along the principal crystal axis. For our numerical settings, the preferred spin direction is parallel to the c-axis; however, we find that the MCA is very small, on the order of 1 μeV, which is the resolution limit of our calculation. In addition, we find the size of the orbital magnetic moment on Ti to be 0.006 μ_B, which is also negligibly small. Consequently, we do not expect the spin-orbit effects to influence the equilibrium magnetic properties significantly.

S1.2 Driven phonon amplitudes

Based on the calculated phonon spectrum, we can simulate the phonon dynamics to estimate the mode amplitudes and atomic displacements induced by a resonant THz pump excitation, as described elsewhere. We model the material as a medium whose dielectric function is given by a sum of Lorentz oscillators (Tab. S1) with phenomenological damping constants, and we solve the equations of motion in response to an electric field pulse, $E(t)$. The field $E(t)$ is taken to be a Gaussian pulse whose center frequency is in resonance with the phonon and whose full width at half maximum (FWHM) matches that of the experiment (roughly 250 fs). For driving field strengths of several MV/cm, as are achieved in our experiment, we estimate that the phonon amplitudes lead to excursions of the oxygen ions within the $YTiO_3$ unit cell of up to tens of pm (see Tab. S2). By comparison, at a temperature of 10 K, mean squared thermal vibration amplitudes are typically on the order of 1 pm or less. Note that the phonon amplitude is linearly proportional to the strength of $E(t)$, so more intense pulses will lead to even larger atomic motions.

Frequency (THz)	$Q_{\mathrm{max,driven}}$ ($\sqrt{\mathrm{u}}$Å)	Max. driven disp. (pm)	RMS therm. disp. (pm)
4.1	3.0	8.3	0.97
9.0	1.2	12.7	2.5
15.9	0.6	4.5	1.4
16.7	0.3	2.1	0.86

Table S2: Comparison between optically driven and thermally activated vibrational motion. $Q_{\mathrm{max,driven}}$ corresponds to the maximum phonon amplitude following a resonant excitation with 5 MV/cm field strength. The third column provides the corresponding maximum displacement of oxygen ions, and the final column shows the equivalent root mean squared thermal displacements.

S1.3 Spin-Phonon Coupling

Utilizing the results for the phonon eigenmodes and magnetic structure, we mapped the spin-phonon interactions. Due to symmetry considerations, polar phonon modes in the centrosymmetric $YTiO_3$ structure can only alter the magnetic exchange quadratically. Consequently, to lowest order, the following Heisenberg Hamiltonian applies for the B_{2u} modes considered in our experiment,

$$E_{\mathrm{mag}} = \sum_k \sum_{ij,(ab)} \left(J_{\mathrm{in}} + \omega_k \lambda_{\mathrm{in},k} Q_k^2\right) \mathbf{S}_i \cdot \mathbf{S}_j + \sum_k \sum_{ij,(c)} \left(J_{\mathrm{out}} + \omega_k \lambda_{\mathrm{out},k} Q_k^2\right) \mathbf{S}_i \cdot \mathbf{S}_j, \qquad (1)$$

where the k sum runs over all B_{2u} phonon modes with frequencies ω_k and amplitudes Q_k, the ij sums run over the nearest neighbor spins either in the ab plane or along the c-axis, and $\lambda_{\mathrm{in},k}$ and $\lambda_{\mathrm{out},k}$ are the coefficients describing the modification of in- and out-of-plane exchanges by the phonon, respectively. To compute these coefficients, we displaced the atoms along the

phonon eigenmode coordinates and reevaluated the exchange interactions, similar to Ref. [57]. In Fig. S1(a), we show the resulting modulation for the B_{2u} phonon mode at 9 THz. For this specific mode, we find that the phonon distortion strongly modifies both the in- and out-of-plane exchange interactions. To summarize the results for all B_{2u} phonons, we show in Fig. S1(b,c,d) the in-plane, the out-of-plane, and the averaged (λ_{avg}) spin-phonon coefficients for all modes. The averaged spin-phonon coefficient weights the individual coefficients by the number of nearest neighbors and is defined as,

$$\lambda_{\text{avg},k} = \frac{2\lambda_{\text{in},k} + \lambda_{\text{in},k}}{3\omega_k} \tag{2}$$

The sign of λ determines whether the exchange interaction will be enhanced or diminished by phonon excitation. For the case of ferromagnetic $YTiO_3$, the equilibrium values of J_{in} and J_{out} are both negative, therefore, if $\lambda < 0$ phonon excitation would enhance the underlying ferromagnetic exchange, while it would be reduced if $\lambda > 0$. (We note that λ_{avg} provides a single number to conveniently quantify the strength of the spin-phonon interaction, but λ_{in} and λ_{out} are needed to fully understand the predicted phonon-induced changes to the magnetic structure.)

From comparing the results in Fig. S1 with the experimental (equilibrium) spin-phonon frequency shifts presented in Extended Data Fig. 10, we can see that the calculations agree reasonably well for the modes studied experimentally in both the sign and relative magnitude. This agreement highlights the accuracy of our calculations in equilibrium and in the linear response, which helped inform our choice of which phonons to excite in our pump-probe experiment. However, crucially, the sign of λ is *opposite* to what we observe experimentally in our non-equilibrium measurements in the main text: for example, the positive λ for the 9 THz mode implies that it would weaken ferromagnetism, whereas we find the strongest pump-induced enhancement when driving that mode. We attribute this disparity to the nonlinear and dynamical nature of the pump-induced response, which is not captured in the DFT calculations and can lead to a significant deviation from the linear spin-phonon response found in equilibrium.

S1.4 Wannier Functions

Lastly, we computed the localized Wannier functions and corresponding tight-binding parameters as a function of the B_{2u} phonon mode amplitude. The results of these calculations are used to determine the crystal field splitting/orbital gap shown in Fig. 4(b) in the main text and used as input for the dynamical model presented in Sec. S2. As a projection basis for the Wannier functions, we consider only the t_{2g} states on Ti. To get an unbiased parameterization, we turned off the Coulomb parameter U and the spin polarization for these calculations.

S1.5 Numerical Settings

We performed our computations with the Vienna ab-initio simulation package, VASP.6.2 [58]. For the phonon calculation, we used the Phonopy software package [55] and for Wannierization, we used the Wannier90 package [59]. Our computations relied on pseudopotentials generated within the Projected Augmented Wave (PAW) [58] method. Specifically, we took the following default potential configurations: Ti $3p^6 4s^1 3d^3$, Y $4s^2 4p^6 5s^2 4d^1$, and O $2s^2 2p^4$. We applied the Local Spin Density Approximation (LSDA) approximation for the exchange-correlation potential, including he Hubbard $U - J$ parameter to account for the localized nature of the Ti d-states with $U - J = 4$ eV. A $9 \times 9 \times 7$ Monkhorst [60] generated k-point-mesh sampling of the Brillouin zone was used with a plane-wave energy cutoff of 600 eV. The self-consistent calculations were iterated until the change in total energy was converged at the level of 10^{-8} eV.

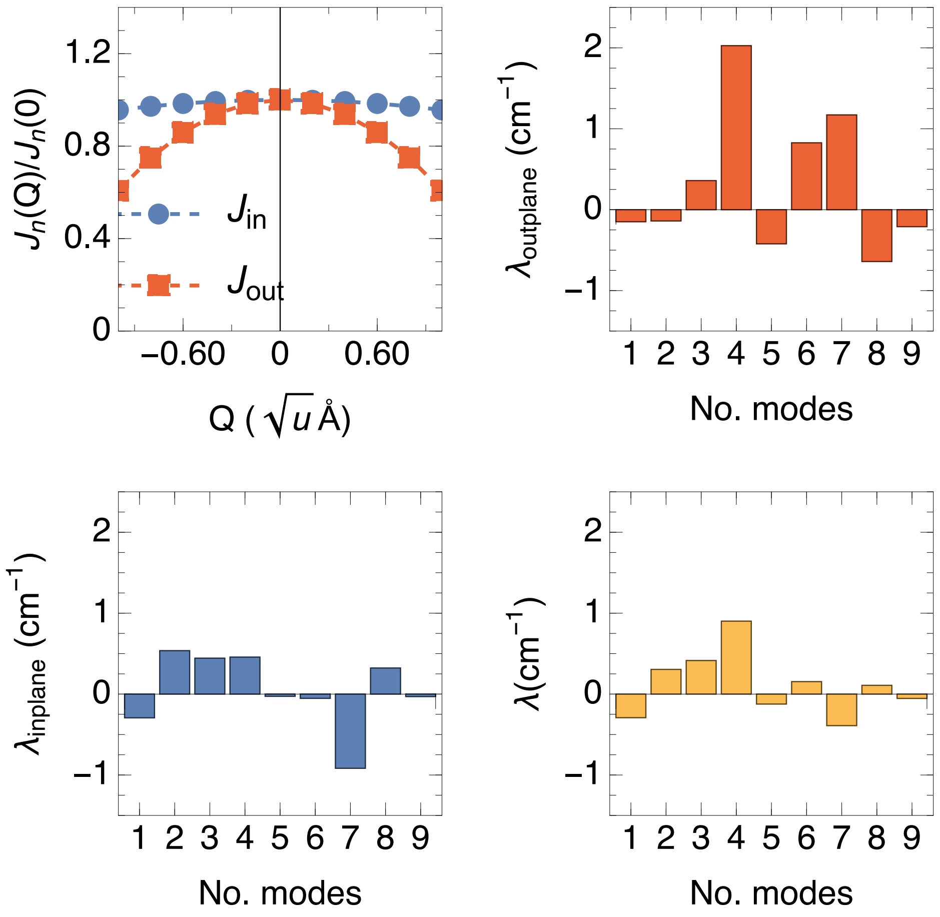

Figure S1: (a) Computed dependence for the exchange interaction as a function of the phonon mode amplitude for the 9 THz B_{2u} polar mode. (b,c) Individual out- and in-plane spin-phonon coupling coefficients for all polar B_{2u} modes obtained from fitting the first-principle computations. (d) Averaged spin-phonon coupling constants computed from (b,c) and Eqn. 2. The modes in (b,c,d) are numbered according to Tab. S1

S1.6 U-dependence

To characterize the robustness of the DFT results, we analyze the dependence of all computed properties as a function of the on-site Hubbard U parameter used in the calculations. In Fig. S2 we show the results of such an analysis on the ground state crystal and electronic structure. Notably, a U of at least 2 eV is needed to make the system insulating, but otherwise we find a smooth, monotonic U-dependence. More importantly, as shown in Fig. S3, for any finite U, the magnetic ground state is a c-axis ferromagnet. The exchange constants maintain the same sign and relative strength over the entire U range with only their overall magnitude varying. This statement is also true for the phonon frequencies and, most important of all, the spin-phonon coupling constants. That is, independent of the chosen U, we find the same discrepancy between the calculated signs of λ and the experimentally observed pump-induced magnetic response. This result provides further evidence that electronic and magnetic dynamics beyond simple adiabatic lattice deformations are important.

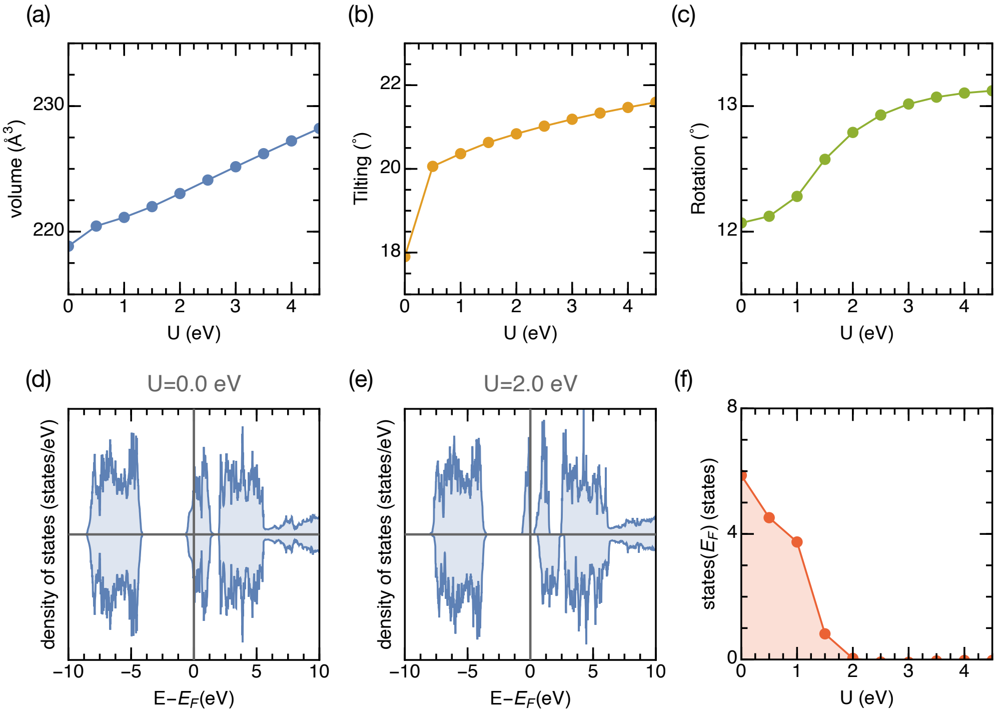

Figure S2: (a) Unit-cell volume, (b) octahedral tilt, and (c) octahedral rotation as a function of U. (d,e) Exemplary total density of states for two distinct values of U. (f) Electron count at the Fermi-level as a function of U.

S2 Magnetization Dynamics

Here, we consider the dynamics of the pump-induced magnetization and provide a calculation to qualitatively demonstrate how the strong drive can lead to an accelerated timescale for longitudinal magnetic relaxation. We explain the presence of two timescales in the MOKE signal by arguing that during the coherent phonon oscillations, a new channel for spin relaxation opens, allowing for rapid growth, or decay, of the Kerr rotation signal, depending on the phonon mode pumped. Then, after the phonon mode has rung-down and no longer exhibits coherent oscillations, the magnetization slowly returns to equilibrium via the conventional pathways for longitudinal magnetization relaxation. In $YTiO_3$, which is a ferromagnetic insulator with weak anisotropy, this timescale can be quite long, explaining the apparent metastability of the pump-induced state, which essentially becomes "trapped" after the phonon oscillations diminish. This process is schematically illustrated in Fig. S4.

S2.1 Equilibrium magnetization lifetime

The longitudinal component of the magnetization is often subject to a bottleneck in its dynamics (the well-known Einstein-de Haas effect) since it is approximately conserved in a ferromagnetic system, with the dominant channel for relaxation often being provided by spin-orbit coupling, which ultimately transfers angular momentum from the spin into the orbital and then lattice degrees of freedom. We, therefore, begin by considering the effect of spin-orbit coupling through the atomic $\mathbf{L}\cdot\mathbf{S}$ coupling on the Ti sites. For the sake of simplicity, we provide only a rough calculation of the orbital dynamics here, and a more detailed treatment will be explored in a forthcoming publication.

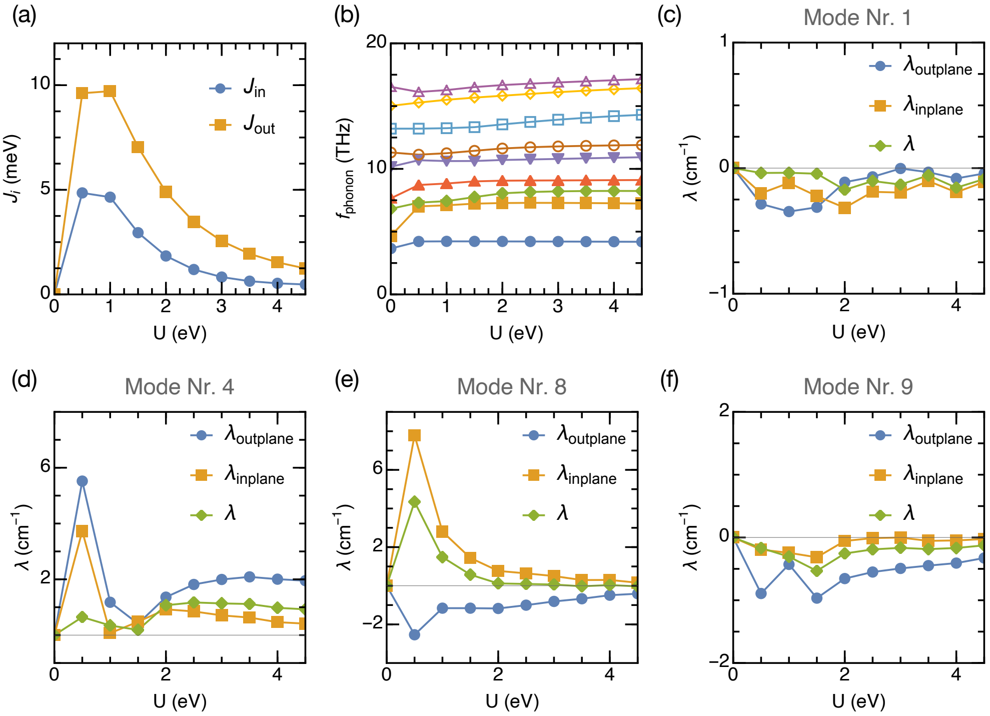

Figure S3: (a) Exchange constants, (b) phonon frequencies of the B_{2u} modes, and (c-f) spin phonon coupling constants for selected B_{2u} modes as a function of U.

The relevant Hamiltonian is taken to be

$$H_{\text{LS}} = \sum_j \lambda \hat{\mathbf{L}}_j \cdot \hat{\mathbf{S}}_j \tag{3}$$

where j runs over all the Ti sites and $\hat{\mathbf{L}}_j$ is the t_{2g} orbital angular momentum operator. In terms of the three t_{2g} levels, the angular momentum operator on site j is given by

$$L_j^l = -i\epsilon_{lmn} \left(|n\rangle\langle m| - |m\rangle\langle n| \right). \tag{4}$$

This is odd under time-reversal and is diagonalized in the spherical eigenbasis of the three levels, which are in reality split by the crystal field. Characteristic values of the atomic spin-orbit coupling for a $3d$ transition metal are $\lambda \sim 10 - 20$ meV [61].

For simplicity, we project the orbital angular momentum onto the lowest crystal field levels, in which case we can write

$$\hat{\mathbf{L}}_j = \mathbf{n}_j \hat{\boldsymbol{\tau}}_j^2, \tag{5}$$

where $\mathbf{n}_j$ is a unit vector which characterizes the matrix elements of the transition between the crystal-field ground-state and first-excited state, and $\hat{\boldsymbol{\tau}}_j^2 = -i|1\rangle_j|\langle 0|_j + \text{h.c.}$ is a second-quantized operator which corresponds to the orbital angular momentum of the lowest two orbitals on the Ti site.

We consider a magnetization oriented along the c axis and determine the transverse spin-wave self-energy due to the coupling to the orbitals in second-order perturbation theory, assuming the orbitals are acting simply as a bath. We focus on the isotropic contribution to the

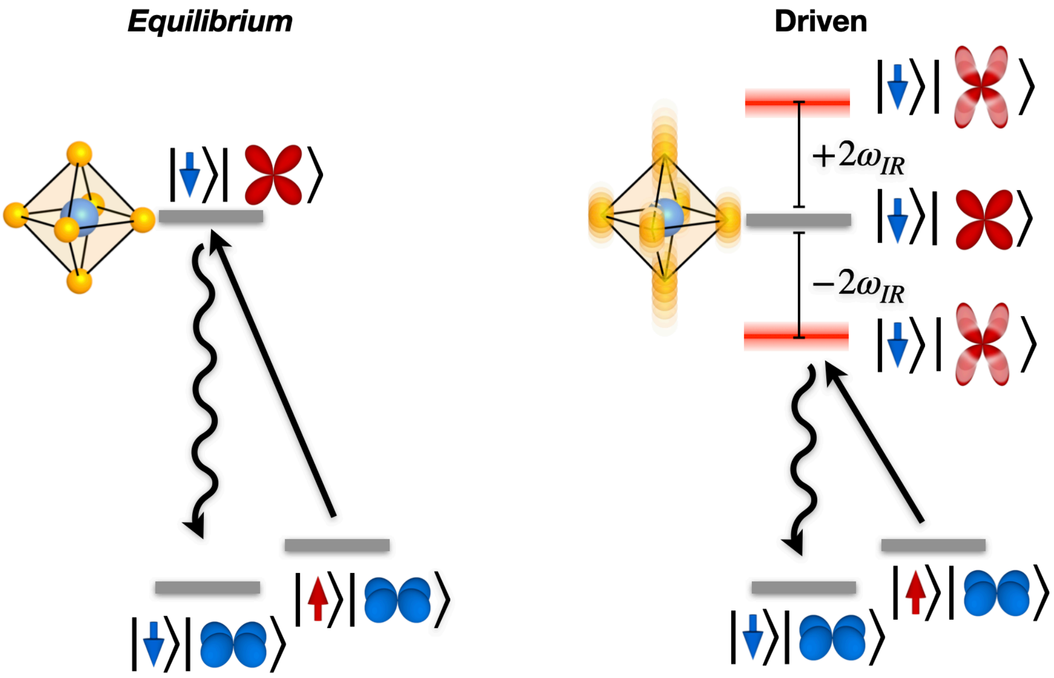

Figure S4: Schematic illustration of the spin flip pathways for modifying the longitudinal magnetization in equilibrium and in the coherently driven state. The spin flip decay proceeds by combined spin-orbital flip via spin-orbit coupling, followed by an orbital decay process which is spin-independent. In equilibrium, this process is intrinsically slow due to the large separation of scales between the crystal field ground state and first excited state (shown schematically as blue and red orbital states). In the driven scenario, the decay time is accelerated due to the strong drive, which distorts the crystal field environment. The drive induces sidebands of the excited orbital state at twice the phonon frequency, which reduces the effective crystal field splitting associated with the orbital flip process. The spin flip decay time is accelerated since the intermediate state is brought closer to resonance. Importantly, the spin flip pathway is enhanced only while there are strong coherent oscillations of the crystal field state, returning to the slower pathway once the oscillations have rung down.

retarded spin-wave propagator (neglecting additional in-plane anisotropies) via local self-energy, averaged over the four Ti sublattice sites,

$$\Sigma^R(t,t') = \frac{1}{2}\lambda^2 \langle\!\langle \frac{\mathbf{n}_j \cdot \mathbf{\Pi}_\perp \cdot \mathbf{n}_j}{2} \rangle\!\rangle \left(\frac{D^R(t,t') + D^A(t',t)}{2} \right), \tag{6}$$

with the retarded orbital correlation function

$$D^R(t,t') = -i\theta(t-t')\langle[\hat{\tau}_j^2(t), \hat{\tau}_j^2(t')]\rangle, \tag{7}$$

and the advanced correlation function follows as $D^A(t,t') = D^R(t',t)^\dagger$. The first term describes the in-plane isotropic projection of the angular momentum anisotropy tensor, averaged over the four Ti sublattices (indicated by the double brackets). This averaging is expected to be acceptable for a long-wavelength, low-energy magnon which will vary little on the atomic scale, and therefore see an effectively averaged potential. For the orbital bath, we take as an approximation for the orbiton spectral function a Lorentzian response with retarded correlation function

$$D^R_{\mathrm{orb}}(\omega) = \frac{2\Delta}{(\omega + i\Gamma)^2 - \Delta^2}, \tag{8}$$

with bare crystal-field splitting taken from *ab initio* calculations in Sec. S1 of $\Delta \sim 140$ meV and orbital linewidth $\Gamma \sim 10$ meV.

The longitudinal magnetization relaxation manifests as the transverse spin-wave lifetime. We find, for the equilibrium case, that the imaginary part of the self-energy goes as $-\Im\Sigma^R(\omega) \sim \alpha_{\mathrm{eq}}\omega$, where α_{eq} is the effective equilibrium decay rate. Because the magnon band is so far detuned below the orbiton resonance, this decay rate is highly suppressed. Based on parameters above we find an estimate of $\alpha_{\mathrm{eq}} \sim \langle\!\langle |\mathbf{n}_\perp|^2 \rangle\!\rangle 2\Gamma\lambda^2/\Delta^3$. For a magnon of frequency $\omega \sim 1$ meV, we find a corresponding lifetime of order 4 ns, which is consistent with the lifetime of the induced magnetization found in experiment (Extended Data Fig. 9).

S2.2 Driven magnetization lifetime

Next, we consider the lifetime in the driven case, where the coherent phonon oscillations induced by the pump modulate the crystal field state in time. The time-dependence of the crystal field state manifests through the eigenvectors $\mathbf{n}_j$. We focus here on the B_{2u} phonon at 9 THz (mode label 4 in Tab. S1). We evaluate the lifetime using the orbital transition matrix eigenvectors $\mathbf{n}_j(t)$ obtained from the *ab initio* calculations in Sec. S1, which acquire time-dependence in the presence of the driven phonon via $\mathbf{n}_j(t) = \mathbf{n}_j + \delta\mathbf{n}_j Q_{IR}^2(t)$, where Q_{IR} is the phonon amplitude. Fig. S5 shows that the changes in the orientation of the orbital angular momentum vector due to the phonon oscillations are quite substantial for this mode (results are similar for other modes). Importantly, because the orbital transition is of Raman character, the coupling is proportional to Q_{IR}^2, which involves oscillations at $\pm 2\Omega_d$.

Most relevantly, the oscillating phonon amplitude impacts the components of the orbital angular momentum perpendicular to the magnetization. leading to a time-dependent transverse field and resultant spin-flip processes. This effect is characterized by time-dependent angular momentum anisotropy tensor $\langle\!\langle \frac{\mathbf{n}_j(t)\cdot\mathbf{\Pi}_\perp\cdot\mathbf{n}_j(t')}{2} \rangle\!\rangle$, which enters in the magnon self energy. We use a simplified model here, parameterizing the drive effects as

$$\langle\!\langle \frac{\mathbf{n}_j(t) \cdot \mathbf{\Pi}_\perp \cdot \mathbf{n}_j(t')}{2} \rangle\!\rangle = \langle\!\langle \frac{\mathbf{n}_j \cdot \mathbf{\Pi}_\perp \cdot \mathbf{n}_j}{2} \rangle\!\rangle \left[1 + I^2 \cos(2\Omega_d(t-t'))\right]/(1+I^2) \tag{9}$$

in terms of the equilibrium projection $\langle\!\langle \frac{\mathbf{n}_j\cdot\mathbf{\Pi}_\perp\cdot\mathbf{n}_j}{2} \rangle\!\rangle \sim .483$ and unitless strength parameter $I \propto Q_{\mathrm{max}}^2$, roughly proportional to the pump fluence. This parameterization discards terms which explicitly break time-translational symmetry, such as $\cos 2\Omega_d(t+t')/2$. In principle, these terms

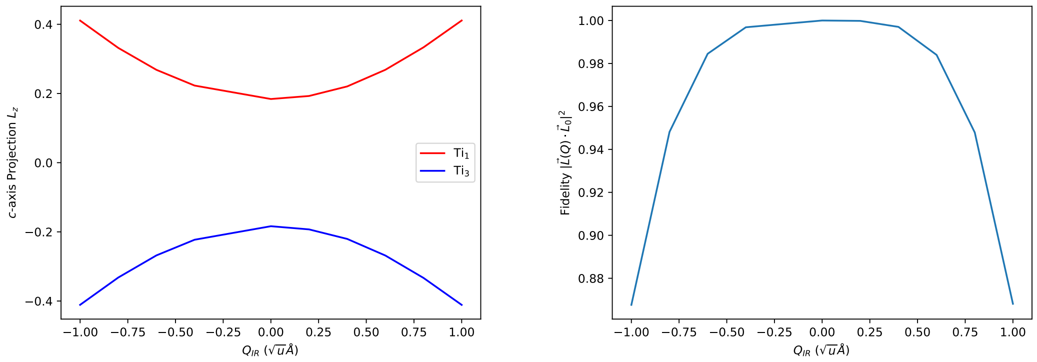

Figure S5: (a) Change in c-axis component of $\mathbf{L}$ on particular Ti sites with respect to the IR phonon displacement. The maximal displacement during the pulse is of order of $Q_{\mathrm{IR}} \sim 1.2$ in these units. (b) Fidelity, measured by angular momentum overlap with equilibrium, as a function of the IR mode displacement, illustrating a dramatic reorientation of the orbital angular momentum during the phonon oscillation. Shown are calculations for mode 4, with a frequency of 9 THz.

lead to a backfolding of the self-energy in frequency space by the drive frequency; capturing these effects requires a full solution to a quantum kinetic equation. This model also preserves the "overall" size of the matrix element squared, such that the spectral weight is merely shifted from the main peak to the (Floquet) sidebands.

The non-equilibrium self-energy at low frequencies can then be written in terms of the equilibrium self-energy (Eq. 7) as

$$\Sigma^R_{\mathrm{neq}}(\omega) = \frac{1}{1+I^2}\Sigma^R(\omega) + \frac{I^2}{1+I^2}\left(\frac{\Sigma^R(\omega+2\Omega_d)+\Sigma^R(\omega-2\Omega_d)}{2}\right). \tag{10}$$

For finite I, this experession leads to an enhancement of the damping rate for magnons (and hence, a shortening of the risetime of the pump-induced change in the magnetization).

We compare the driven and equilibrium relaxation rates as a function of frequency for a few different estimates of the fluence parameter I in Fig. S6. Even within this relatively crude model, one can see that the magnetic relaxation rate due to spin-orbit coupling can be dramatically enhanced in the pumped state. The enhancement is due to the pump introducing sidebands, which increase the spectral overlap of the magnon states with the bath, allowing for a more efficient decay of the spin (*i.e.* faster angular momentum transfer). The change in the magnetization lifetime in the driven case, relative to that in equilibrium, is shown in Fig. S7 as a function of the model parameter I. We find a dramatic reduction in the lifetime

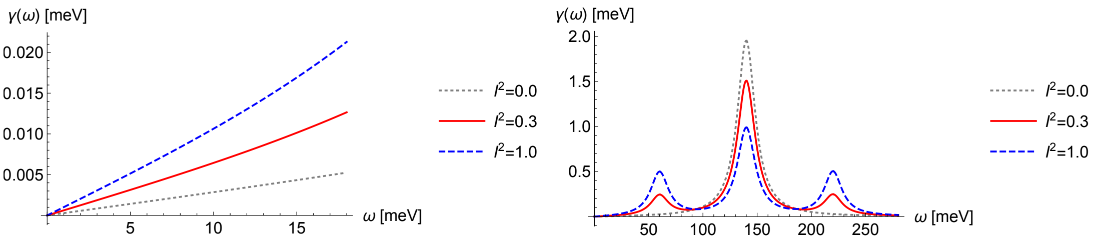

Figure S6: (a) Dependence of magnon relaxation rate $\gamma(\omega)$ on frequency for different pump strengths, modeled by parameter I. Equilibrium (gray, dotted) has very small spectral weight at the magnon frequencies, while the modest pump (red, solid) and strong pump (blue, dashed) scenarios have increasing spectral weight at low frequencies. (b) Same functions, plotted over the range of $[0, 2\Delta_{\mathrm{CF}}]$, illustrating how the pump produces sidebands which enhance the overlap at low frequencies.

for a sufficiently strong pump, which corresponds to a faster risetime of the non-equilibrium magnetization in the experiment.

These calculations provide a plausible explanation for the experimental observation that the time scales of the magnetization growth into the non-equilibrium state and its decay back to equilibrium are divergent. To summarize the argument, the resonant THz frequency pump excites a particular phonon mode, which rings down after the THz pulse is gone with a damping rate given by the inverse lifetime of the phonon. During this phonon lifetime there is an enhancement in the transfer of orbital angular momentum via the mechanism described in this section, which allows the magnetization to change rapidly to reach the non-equilibrium state. Once the phonon drive has subsided, this enhanced decay pathway is no longer present, leaving the system in a metastable non-equilibrium magnetic state. Eventually, it returns to its equilibrium magnetization through the relatively slow, undriven, magnetization relaxation process. This theoretical model is supported by the observation that the MOKE signal risetime appears to be bounded by the phonon lifetime (see Extended Data Fig. 8 in the main text), indicating that the transfer of angular momentum happens rapidly only during the coherent oscillations.

Although the treatment presented here can qualitatively explain our experimental observations, We believe a more detailed investigation of these effects is warranted, especially due to the interesting and novel nature of this dynamical route. In particular, we think it will be important to include (i) the in-plane anisotropy, which also experiences dramatic changes during the pump (and leads to anomalous magnon correlation function), and (ii) the finite pump effects and terms which break time-translation symmetry (which leads to a full back folding of the Floquet spectrum). These aspects will be pursued in a follow up theoretical investigation.

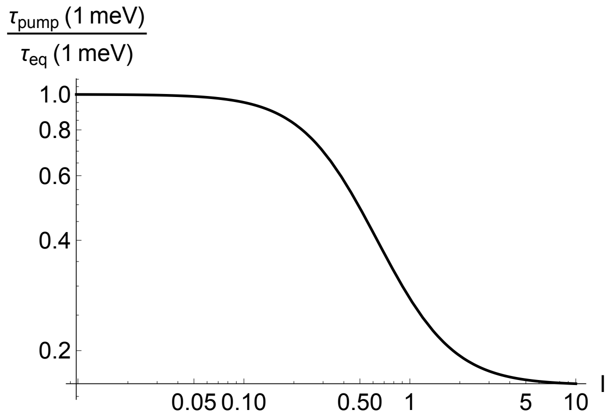

Figure S7: Calculated ratio of the lifetime ($\tau = 1/\gamma(\omega)$) in the driven case to the lifetime in the equilibrium case for a magnon of frequency $\omega = 1$ meV as a function of the pump strength, illustrating an acceleration of much greater than 5-fold is possible for sufficient pump strength.

References

[54] N. Marzari and D. Vanderbilt. Maximally localized generalized Wannier functions for composite energy bands. *Phys. Rev. B*, 56:12847, 1997.

[55] A. Togo and I. Tanaka. First principles phonon calculations in materials science. *Scr. Mater.*, 108:1, 2015.

[56] M. Gu and J.M. Rondinelli. Nonlinear phononic control and emergent magnetism in Mott insulating titanates. *Phys. Rev. B*, 98:024102, 2018.

[57] M. Fechner, A. Sukhov, L. Chotorlishvili, C. Kenel, J. Berakdar, and N.A. Spaldin. Magnetophononics: Ultrafast spin control through the lattice. *Phys. Rev. Mat.*, 2:064401, 2018.

[58] G. Kresse and D. Joubert. From ultrasoft pseudopotentials to the projector augmented-wave method. *Phys. Rev. B*, 59:1758, 1999.

[59] A.A. Mostofi, J.R. Yates, G. Pizzi, Y.S. Lee, I. Souza, D. Vanderbilt, and N. Marzari. An updated version of wannier90: A tool for obtaining maximally-localised Wannier functions. *Comput. Phys. Commun.*, 185:2309, 2014.

[60] H. Monkhorst and J.D. Pack. Special points for Brillouin-zone integrations. *Phys. Rev. B*, 13:5188, 1976.

[61] I.V. Solovyev. Superexchange interactions in orthorhombically distorted titanates $R\mathrm{TiO}_3$ (R = Y, Gd, Sm, and La). *New J. Phys.*, 11:093003, 2009.
